# Global, regional, and national burden of stroke attributable to diet high in sodium from 1990 to 2019: a systematic analysis from the global burden of disease study 2019

**DOI:** 10.3389/fneur.2024.1437633

**Published:** 2024-08-14

**Authors:** Xuan Zhang, Wen-qian Ye, Xue-Ke Xin, Ying-jie Gao, Fan Yang

**Affiliations:** ^1^The Fifth Clinical Medical College of Shanxi Medical University, Taiyuan, China; ^2^Shanxi Provincial People's Hospital, Taiyuan, Shanxi, China; ^3^School of Pharmaceutical Science, Medicinal Basic Research Innovation Center of Chronic Kidney Disease, Ministry of Education, Shanxi Medical University, Taiyuan, Shanxi, China; ^4^School of Management, Shanxi Medical University, Taiyuan, Shanxi, China

**Keywords:** GBD 2019, mortality, disability-adjusted life year, stroke, diet high in sodium

## Abstract

**Purpose:**

Given the increasing occurrence of stroke and high-sodium diets (DHIS) over the past 30 years, it is crucial to assess the global, national, and regional impact of DHIS on the burden of stroke.

**Methods and materials:**

The Global Burden of Diseases Study 2019 provided the study's data. We used the Bayesian meta-regression tool DisMod-MR 2.1 to evaluate the burden of stroke attributable to DHIS. Age-standardized disability-adjusted life years (ASDR) and age-standardized mortality rate (ASMR) were used to quantify the burden. We perform correlation analysis utilizing the Spearman rank-order correlation method, and we calculate the estimated annual percentage change (EAPC) to evaluate temporal trends.

**Results:**

Globally, DHIS accounts for 17,673.33 thousand disability-adjusted life years (DALYs) and 700.98 thousand deaths of stroke in 2019. The burden of stroke attributable to DHIS has declined between 1990 and 2019 globally and in the majority of regions, with the largest declines seen in regions with high sociodemographic indexes (SDI). Both ASMR and ASDR were higher regionally in regions with moderate SDI than those in developed regions. Furthermore, the absolute values of EAPC, reflecting the rate of decrease, were notably lower in these regions compared to developed nations. High-income North America, categorized within the SDI regions, notably witnessed the smallest decline in ASDR over the last three decades. Additionally, from 1990 to 2019, males consistently bore a larger burden of stroke attributable to DHIS.

**Conclusion:**

The burden of stroke attributable to DHIS remained a major concern despite advancements in public knowledge of stroke and their utilization of emergency medical services. Over the past 30 years, more burden has been placed on males and regions with moderate SDI values; in males, higher EAPC values for both ASMR and ASDR have been found. This underscores the urgent need for effective interventions to alleviate the burden of stroke associated with DHIS.

## 1 Introduction

Stroke, a cerebrovascular disorder characterized by the sudden interruption of blood flow to the brain, continues to pose a significant burden on global health, contributing to both morbidity and mortality (1). According to the Global Burden of Disease Study (GBD) 2019, stroke maintained its status as the second-leading cause of death and the third-leading cause of disability combined, as measured by disability-adjusted life years (DALYs), in 2019 (2). These findings are consistent with the results from the GBD 2017 (3). While advances in stroke treatment have improved outcomes, effective prevention strategies, and optimized rehabilitation interventions are essential for reducing the burden of stroke-related disabilities (4). While the prevalence of high body mass index emerged as the fastest-growing risk factor for stroke between 1990 and 2019, concerted efforts to mitigate this risk through prevention measures have been widely adopted worldwide (5). However, the burden of stroke attributable to diet high in sodium (DHIS) has not received commensurate attention (5). Despite its significant impact on stroke incidence and outcomes, DHIS remains an under-addressed risk factor.

The World Health Organization (WHO) recommends a daily sodium intake of <2,000 mg over a 24-h period to prevent chronic diseases (6). Clear evidence indicates that high sodium intake is associated with various health issues, including cardiovascular diseases (CVDs) and other chronic diseases (7). DHIS is associated with elevated blood pressure (BP), thereby increasing the mortality risk of CVDs in adults, irrespective of hypertension status (8). He et al. (9) demonstrated that reducing salt intake for more than 4 weeks significantly lowered BP, highlighting the potential benefits of sodium reduction. Similarly, Graudal et al. (10) observed a significant relationship between sodium reduction and BP, particularly among individuals in the highest 25^th^ percentile of the population. Moreover, Gupta et al. also found that dietary sodium reduction significantly lowered BP in the majority of middle-aged to elderly adults in a crossover trial which included 213 individuals aged 50 to 75 years from different races (11).

GBD 2019 indicates that approximately 143 million DALYs and 6 million deaths worldwide are attributable to DHIS (2). While other previous GBD studies have effectively quantified the prevalence and attributable burden of stroke, these findings were primarily based on data from specific countries, limited database or encompassed all risk factors (5, 12–15). As a result, the global burden of stroke specifically attributable to DHIS remains inadequately elucidated. The GBD 2019 systematically reviewed and integrated data from 84 risk factors (2, 16), presenting an opportunity to examine the epidemiology of stroke attributable to DHIS at global, regional, and national levels. Mortality and DALYs attributed to DHIS in 24 regions, as well as 204 countries and territories, were analyzed from 1990 to 2019 to assess trends in the burden of stroke associated with DHIS (2, 16). The study also examined correlations between the burden of stroke attributable to DHIS and risk factors such as sex, age, and disparities in economic development.

## 2 Materials and methods

### 2.1 Data resource and disease definition

The data utilized in this research was extracted from the GBD 2019 database, which is accessible at http://ghdx.healthdata.org/gbd-results-tool, to assess the burden of stroke. Our sources for clinical data on stroke encompassed hospital records, emergency department records, insurance claims, surveys, and vital registration systems from various regions globally. The methodology for data input, mortality estimation, and modeling for GBD 2019 has been thoroughly demonstrated in previously published research articles (2, 3, 5, 16). Our research focuses on the burden of stroke associated with DHIS from 1990 to 2019 in 204 countries and territories. The definition of stroke employed in this study adheres to the criteria outlined in the International Statistical Classification of Diseases and Related Health Problems, 10^th^ Revision (ICD-10). It encompasses corresponding diagnostic codes within this classification: I63–I63.9, I65–I66.9, I67.2–I67.3, I67.5–I67.6, I69.3, I60–I62.9, I67.0–I67.1, I68.1–I68.2, and I69.0–I69.2 (2, 16). Additionally, in line with the parent GBD risk factor study, DHIS was defined as average 24-h urinary sodium excretion (in grams per day) greater than the Theoretical Minimum-Risk Exposure Level (TMREL), which was 1–5 grams (16).

### 2.2 Socio-demographic index

The burden of stroke attributable to DHIS was calculated in relation to the level of development at the country level, as constrained by the socio-demographic index (SDI) (2, 16), The burden of stroke attributable to DHIS was assessed relative to the country's level of development, as constrained by the SDI. SDI was defined as a composite indicator that integrates three distinct metrics: lag-distributed income per capita, average educational attainment for individuals aged 15 years and older, and the total fertility rate among individuals aged younger than 25 years. Based on this criterion, the 204 countries and territories could be divided into five separate groups according to their SDI values: low SDI (<0.45), low-middle SDI (≥0.45 and <0.61), middle SDI (≥0.61 and <0.69), high-middle SDI (≥0.69 and <0.80), and high SDI (≥0.80).

### 2.3 Risk factors

The GBD 2019 risk factor calculation process used a six-step comparative risk assessment framework. Initially, risk-outcome pairs were identified, incorporating only those outcomes meeting the criteria of convincing or plausible evidence set by the World Cancer Research Fund (16). Secondly, the relative risk (RR), representing the relationship between exposure and each risk-outcome pair, was estimated. For the purpose of to estimate RRs, meta-analyses of published systematic studies were performed; GBD 2019 contained 81 new systematic reviews. Thirdly, the mean levels of risk exposure were determined by examining government statistics, published studies, household surveys, and censuses in order to approximate the distribution of risk exposure. Fourthly, the TMREL was established, the dietary TMRELs were updated for GBD 2019 and TMREL was set to zero for harmful dietary risks except for sodium intake, as moderate sodium intake is essential. According to GBD 2019 the TMREL for DHIS was set at 1–5 grams average 24-h urinary sodium excretion. Fifth, the attributable burden and population attributable fraction (PAF) were measured by the GBD study. PAF refers to the percentage of disease burden that may be avoided if a given risk factor's TMREL exposure were reached. It was calculated using the following formula: $PAF=\frac{\int_{x=l}^{m}RR\left(x\right)dx-RR\left(x\right)TRMEL}{\int_{x=l}^{m}RR\left(x\right)P\left(x\right)dx}$, the minimum exposure level was represented by 'l,' and the maximum exposure level by 'm in this formula. Although a dose-response curve of relative risks was used to characterize the risk curve for all dietary risks based on the most recent epidemiological data and newly developed methodologies for GBD 2019, we proceeded to calculate the impact of salt on cardiovascular disease by considering its influence on systolic blood pressure. Finally, the GBD study estimated the PAF and attributable burden for combinations of risk factors. The methodology for these steps has been systematically demonstrated in previous GBD research (16).

### 2.4 Statistical analysis

In this work, we evaluated and compared the stroke mortality and DALY rates at the regional and national levels using the age-standardized mortality rate (ASMR) and the DALYs rate (ASDR), along with their respective 95% uncertainty intervals (95% UI). These rates were adjusted for standardized age structures and demographic characteristics, providing a comprehensive understanding of the epidemiology of stroke associated with DHIS, which aligns with previous published articles (16). As a way to delve into trends over time, the study applied age-standardized rates (ASRs) to determine the expected annual percentage change (EAPC) in both the ASMR and ASDR from 1990 to 2019. There was a 95% confidence interval (95% CI) for each statistic. The following formula, which represents a linear relationship between ASRs and time, was used to estimate the EAPC: y = a + bx + e. The calendar year is represented by x, the regression coefficient is denoted by b, and log10 (ASR) is represented by y in this model. Using this model, the EAPC was computed as follows: EAPC = 100 ^*^ (10∧b - 1). A lower boundary of the 95% CI above zero denotes an increasing trend, while a lower boundary below zero denotes a steady trend when evaluating ASMR and ASDR trends. Conversely, ASMR or ASDR is said to have a stable trend if the lower border is below zero. The anticipated values of ASMR and ASDR within each SDI unit were estimated using Gaussian process regression with a Loess smoother. Next, the association between SDI and ASMR and ASDR was evaluated using Spearman's rank-order correlation. R software (version 4.1.0; https://cran.r-project.org) was used for all statistical analysis, with a *p*-value <0.05 being deemed statistically significant.

## 3 Results

### 3.1 Global, regional and national burden of stroke attributable to DHIS from 1990 to 2019

**Figure 1:**
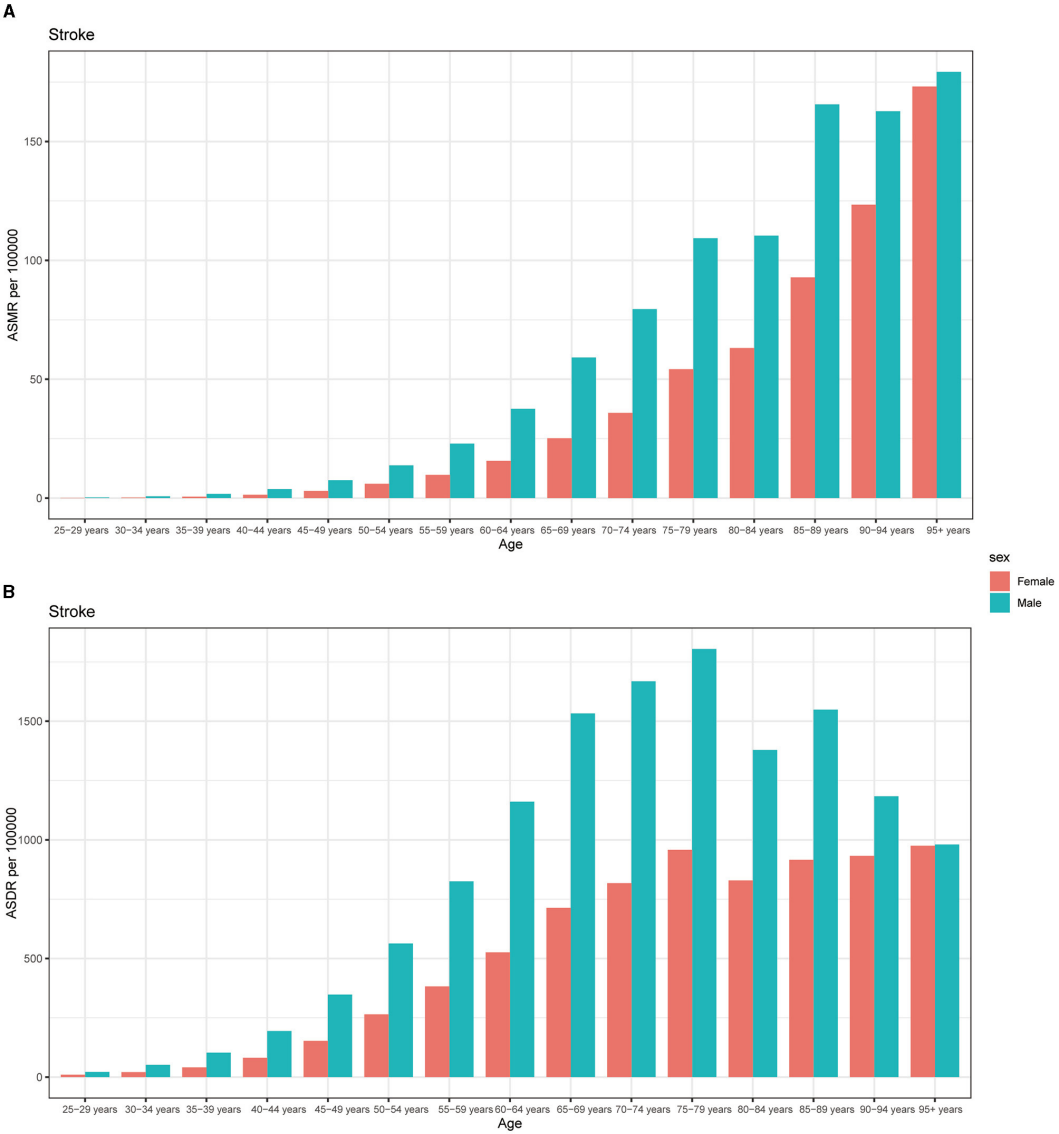
Global age standardized mortality rate of stroke attributable to diet high in sodium. **(A)** The all-cause ASMR per 100,000 associated with stroke attributable to diet high in sodium, for both sexes in 204 countries and territories in 2019. **(B)** The EAPC of ASMR of stroke attributable to diet high in sodium, for both sexes from 1990 to 2019, in 204 countries and territories. ASMR, age standardized mortality rate; EAPC, estimated annual percentage change.

Globally, from 556.26 thousand (95% UI: 193.71 to 1,068.68) in 1,990 to 700.98 thousand (95% UI: 203.31 to 1,448.1) in 2019, the number of stroke deaths attributable to DHIS increased dramatically. But throughout the same time span, the comparable ASMR dropped from 14.76 per 100,000 population (95% UI: 4.88 to 28.98) to 8.69 per 100,000 population (95% UI: 2.47 to 18.07). East Asia (19.83; 95% UI: 7.39 to 35.81) had the highest ASMR at the regional level. On the other hand, Table 1 shows that Australasia had the lowest ASMR (1.39; 95% UI: 0.14 to 5.39). Looking at specific nations, it is clear that the majority of those with high ASMR were found in Latin America and East and Southeast Asia. The country with the highest ASMR was North Macedonia (43.98; 95% UI: 13.86 to 82.19). East Asian and Southeast Asian nations had greater ASMR rates in 2019 overall, with a few notable exceptions being India (3.98; 95% UI: 0.32 to 11.5) and Japan (2.62; 95% UI: 0.32 to 6.01). Spain (0.61; 95% UI: 0.08 to 2.21) and Australia (0.51; 95% UI: 0.06 to 2.07) were the next closest countries with lower ASMR, after Lebanon (0.5; 95% UI: 0.09 to 2.13) (Figure 1A, Supplementary Table 1S).

**Table 1:** Global and regional number of deaths and age-standardized morality of stroke attributable to diet high in sodium for both sexes combined in 1990 and 2019, and EAPC of ASMR from 1990 to 2019.

	**Deaths number (×1,000) in 1990**	**Deaths number (×1,000) in 2019**	**ASMR in 1990**	**ASMR in 2019**	**EAPC 1990–2019**
Global	556.26 (193.71 to 1,068.68)	700.98 (203.31 to 1,448.1)	14.76 (4.88 to 28.98)	8.69 (2.47 to 18.07)	−1.95 (−2.06 to −1.83)
Gender					
Male	327.09 (133.03 to 592.76)	447.18 (149.41 to 859.06)	19.18 (7.35 to 35.4)	12.05 (3.87 to 23.43)	−1.67 (−1.79 to −1.56)
Female	229.17 (62.56 to 489.31)	253.81 (47.16 to 597.86)	11.17 (2.97 to 24.1)	5.79 (1.07 to 13.63)	−2.47 (−2.6 to −2.35)
SDI					
High SDI	57.89 (14.78 to 131.03)	42.89 (7.1 to 109.41)	6.13 (0.36 to 16.41)	2.06 (0.36 to 5.15)	−3.91 (−4.11 to −3.7)
High-middle SDI	189.14 (72.4 to 353.11)	203.98 (67.68 to 398.37)	16.63 (3.15 to 35.83)	10.1 (3.35 to 19.78)	−2.41 (−2.6 to −2.21)
Middle SDI	222.5 (87.79 to 395.36)	314.4 (102.75 to 601.57)	24.1 (6.39 to 46.35)	13.37 (4.12 to 26.08)	−1.92 (−2.04 to −1.79)
Low-middle SDI	67.41 (17.29 to 149.51)	111.05 (22.57 to 262.74)	16.2 (3.13 to 34.82)	8.65 (1.64 to 20.79)	−1.22 (−1.31 to −1.12)
Low SDI	19.15 (1.36 to 55.62)	28.43 (1.59 to 86.31)	14.35 (4.65 to 28.67)	6.28 (0.36 to 19.08)	−1.31 (−1.33 to −1.29)
Region					
Andean Latin America	0.89 (0.04 to 2.46)	1.23 (0.07 to 3.46)	4.67 (0.21 to 12.8)	2.25 (0.12 to 6.37)	−2.6 (−2.85 to −2.35)
Australasia	0.31 (0.03 to 1.2)	0.31 (0.03 to 1.26)	1.39 (0.14 to 5.39)	0.58 (0.06 to 2.27)	−3.35 (−3.52 to −3.18)
Caribbean	1.03 (0.05 to 3.46)	1.59 (0.08 to 5.33)	4.23 (0.21 to 14.03)	3.06 (0.16 to 10.27)	−1.17 (−1.28 to −1.05)
Central Asia	6.94 (1.35 to 14.94)	6.33 (0.55 to 16.82)	16.2 (3.13 to 34.82)	10.79 (0.93 to 28.67)	−1.84 (−2.16 to −1.52)
Central Europe	43.67 (18.87 to 72.06)	33.12 (10.47 to 60.5)	31.81 (13.4 to 53.05)	14.73 (4.57 to 26.68)	−3.15 (−3.33 to −2.97)
Central Latin America	3.67 (0.44 to 9.16)	6.57 (0.75 to 16.36)	4.87 (0.56 to 12.06)	2.87 (0.33 to 7.21)	−2.19 (−2.32 to −2.06)
Central Sub-Saharan Africa	0.81 (0.06 to 3.64)	1.55 (0.1 to 7.1)	4.28 (0.33 to 18.83)	3.56 (0.25 to 15.67)	−0.73 (−0.81 to −0.65)
East Asia	304.91 (138.83 to 507.99)	396.4 (155.07 to 693.34)	37.95 (15.71 to 66.45)	19.83 (7.39 to 35.81)	−2.25 (−2.41 to −2.08)
Eastern Europe	23.96 (2.13 to 68.54)	22.44 (2.04 to 63.95)	9 (0.81 to 26.06)	6.53 (0.6 to 18.51)	−1.93 (−2.48 to −1.37)
Eastern Sub-Saharan Africa	12.08 (0.96 to 30.3)	14.73 (0.73 to 41.2)	18.02 (1.49 to 44.97)	10.98 (0.54 to 29.7)	−1.88 (−1.95 to −1.8)
High–income Asia Pacific	26.23 (7.51 to 49.02)	16.35 (2.38 to 37.65)	14.1 (3.88 to 27.13)	3.06 (0.47 to 6.85)	−5.9 (−6.17 to −5.63)
High–income North America	5.61 (0.4 to 19.16)	8.62 (0.59 to 26.58)	1.55 (0.11 to 5.26)	1.32 (0.09 to 3.98)	−0.79 (−0.96 to −0.62)
North Africa and Middle East	3.04 (0.52 to 12.3)	5.42 (1.01 to 21.35)	1.95 (0.38 to 8.03)	1.37 (0.29 to 5.43)	−1.25 (−1.28 to −1.21)
**Oceania**	0.31 (0.04 to 0.8)	0.68 (0.06 to 1.73)	13.62 (1.79 to 32.93)	11.99 (1.08 to 30.03)	−0.62 (−0.71 to −0.53)
South Asia	30.98 (2.08 to 91.76)	61.64 (4.57 to 174.35)	6.25 (0.41 to 18.66)	4.65 (0.34 to 13.35)	−0.95 (−1.08 to −0.82)
Southeast Asia	58.3 (17.2 to 107.86)	90.75 (15.85 to 188.84)	24.88 (6.8 to 47.54)	16.2 (2.69 to 34.5)	−1.45 (−1.53 to −1.36)
Southern Latin America	2.79 (0.16 to 7.54)	2.5 (0.13 to 6.78)	6.38 (0.37 to 17.12)	2.95 (0.15 to 8)	−2.91 (−3.1 to −2.72)
Southern Sub-Saharan Africa	1.04 (0.05 to 3.87)	1.46 (0.1 to 5.94)	3.88 (0.21 to 14.91)	2.89 (0.21 to 11.75)	−0.98 (−1.38 to −0.58)
Tropical Latin America	7.94 (0.46 to 21.1)	8.88 (0.58 to 23.35)	9.61 (0.57 to 25.52)	3.78 (0.25 to 9.96)	−3.42 (−3.52 to −3.31)
Western Europe	17.31 (1.4 to 56.67)	12.22 (1 to 41.41)	2.95 (0.24 to 9.64)	1.16 (0.09 to 3.77)	−3.51 (−3.74 to −3.28)
Western Sub-Saharan Africa	4.43 (0.19 to 17.09)	8.21 (0.33 to 30.06)	5.72 (0.26 to 22.18)	5.07 (0.22 to 18.47)	−0.33 (−0.39 to −0.26)

ASMR, age-standard morality rate; EAPC, estimated annual percentage change.

In order to study the impact of DHIS on the incidence of stroke, a trend analysis was carried out spanning the years 1990 to 2019. Globally, the data showed consistent trends for both genders, males and females. Surprisingly, these studies showed a noteworthy decline in the burden of stroke attributable to DHIS, particularly in women (−2.47; 95% CI: −2.6 to −2.35) (Table 1). Between 1990 and 2019, a significant reduction in ASMR for stroke attributed to DHIS was observed across all regions. The most substantial decrease was noted in High-income Asia Pacific (−5.9; 95% CI: −6.17 to −5.63). In contrast, the most modest decrease was observed in Western Sub-Saharan Africa (−0.33; 95% CI: −0.39 to −0.26). Additionally, while regions with higher SDI values generally exhibited lower ASMRs, the EAPC values for regions categorized as High and High-middle SDI were also comparatively lower than those of other regions (Table 1). Similar to the trends observed at the regional level, the majority of countries witnessed a decline in the ASMR for stroke attributed to DHIS from 1990 to 2019. However, there were still 22 countries where an increasing burden was noted. Conversely, among the countries experiencing a decreased ASMR over the same period, the most significant decline was seen in the Republic of Korea (−6.59; 95% CI: −6.9 to −6.29), which underscore the diverse trends in the burden of stroke attributed to DHIS across different countries (Figure 1B, Supplementary Table 1S).

**Figure 2:**
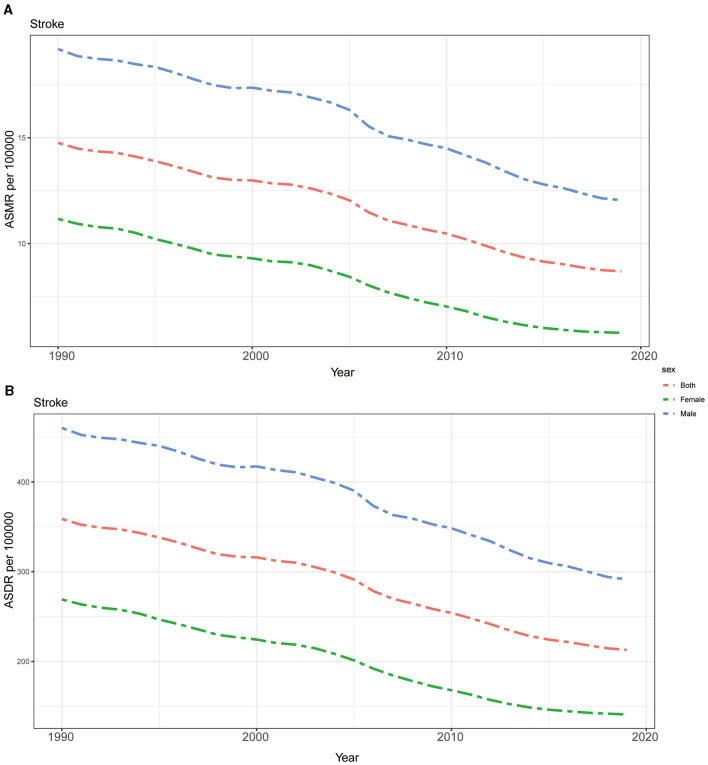
Global age standardized DALYs rate of stroke attributable to diet high in sodium. **(A)** The all-cause ASDR per 100,000 associated with stroke attributable to diet high in sodium, for both sexes in 204 countries and territories in 2019. **(B)** The EAPC of ASDR of stroke attributable to diet high in sodium, for both sexes from 1990 to 2019, in 204 countries and territories. DALYs, disease adjusted life year. ASDR, age standardized DALYs rate; EAPC, estimated annual percentage change.

Globally, from 14,603.67 thousand (95% UI: 5,551.58 to 27,078.55) in 1990 to 17,673.33 thousand (95% UI: 5,751.8 to 34,921.81) in 2019, the number of stroke-related DALYs attributable to DHIS increased. Interestingly, the matching ASDR did not change in the same way as the population; instead, it fell from 358.72 (133.18 to 670.89) per 100,000 population in 1,990 to 212.81 (68.41 to 422.75) per 100,000 population in 2019. At the regional level, it is noteworthy that the ASDR for stroke is higher in regions with intermediate SDI levels. East Asia had the highest ASDR globally, at 480.97 (95% UI: 211.31 to 801.68). In contrast, developed regions generally exhibited lower ASDRs compared to the aforementioned regions. The lowest ASDR was observed in Australasia at 13.19 (95% UI: 1.01 to 47.61) (Table 2). The trend of ASDR mirrored that at the regional level, with developing countries bearing a heavier burden of stroke attributable to DHIS (Figure 2A, Supplementary Table 2S). More specifically, North Macedonia (710.24; 95% UI: 222.92 to 1,311.81) faced the highest ASDR of stroke attributable to DHIS. In contrast, the lowest ASDR were observed in Australia (11.69; 95% UI: 0.96 to 43.22) (Figure 2A, Supplementary Table 2S).

**Table 2:** Global and regional number of DALYs and age-standardized DALYs rate of stroke attributable to diet high in sodium for both sexes combined in 1990 and 2019, and EAPC of ASMR from 1990 to 2019.

	**DALYs number (×1,000) in 1990**	**DALYs number (×1,000) in 2019**	**ASDR in 1990**	**ASDR in 2019**	**EAPC 1990–2019**
Global	14,603.67 (5,551.58 to 27,078.55)	17,673.33 (5,751.8 to 34,921.81)	358.72 (133.18 to 670.89)	212.81 (68.41 to 422.75)	−1.92 (−2.02 to −1.82)
Gender					
Male	8,841.42 (3,815.88 to 15,567.33)	11,527.36 (4,314.22 to 21,291.63)	460.17 (192.56 to 822.41)	291.31 (106.8 to 539.34)	−1.64 (−1.74 to −1.53)
Female	5,762.25 (1,809.42 to 11,820.15)	6,145.97 (1,355.03 to 13,683.49)	269.06 (83.5 to 555.11)	140.88 (31.1 to 313.79)	−2.45 (−2.57 to −2.34)
SDI					
High SDI	1,299.89 (365.9 to 2,799.91)	916.21 (166.56 to 2,241.7)	126.75 (36.2 to 272.26)	51.37 (9.77 to 124.22)	−3.59 (−3.77 to −3.4)
High-middle SDI	4,760.8 (1,977.89 to 8,544.24)	4,942.6 (1,900.81 to 8,992.42)	439.38 (180.09 to 796.44)	243.21 (93.9 to 444.29)	−2.31 (−2.5 to −2.13)
Middle SDI	6,114.93 (2,637.17 to 10,439.84)	8,131.93 (2,950.08 to 14,782.02)	566.47 (230.57 to 985.89)	317.52 (110.95 to 585.75)	−1.98 (−2.08 to −1.88)
Low-middle SDI	1,894.75 (535.26 to 4,057.5)	2,926.36 (650.18 to 6,693.38)	297.59 (79.92 to 650.38)	207.16 (44.51 to 477.71)	−1.26 (−1.34 to −1.18)
Low SDI	529.37 (35.42 to 1,538.53)	750.46 (40.82 to 2,263.58)	213.65 (14.75 to 612.81)	141.37 (7.79 to 424.34)	−1.47 (−1.51 to −1.42)
Region					
Andean Latin America	23 (1.04 to 64.06)	28.93 (1.53 to 82.39)	106.8 (4.8 to 295.07)	50.73 (2.68 to 143.18)	−2.68 (−2.93 to −2.44)
Australasia	6.89 (0.58 to 25.3)	6.05 (0.51 to 22.6)	30.1 (2.51 to 111.2)	13.19 (1.01 to 47.61)	−3.1 (−3.29 to −2.91)
Caribbean	23.04 (1.16 to 79.51)	34.52 (1.76 to 118.72)	88.42 (4.43 to 305.66)	66.77 (3.41 to 229.83)	−0.99 (−1.14 to −0.83)
Central Asia	163.06 (31.75 to 352.24)	154.81 (13.8 to 413.23)	347.94 (67.54 to 756.43)	212.14 (18.91 to 570.71)	−2.19 (−2.47 to −1.9)
Central Europe	935.45 (407.14 to 1,522.03)	594.18 (190.1 to 1,076.78)	643.07 (277.99 to 1,045.11)	277.43 (88.78 to 501.12)	−3.44 (−3.62 to −3.26)
Central Latin America	93.89 (11.41 to 231.61)	152.62 (17.14 to 384.25)	107.81 (12.8 to 269)	63.98 (7.14 to 160.71)	−2.15 (−2.28 to −2.01)
Central Sub-Saharan Africa	23.49 (1.64 to 105.68)	43.62 (2.76 to 194.62)	97.85 (7.22 to 442.44)	77.93 (5.22 to 353.61)	−0.87 (−0.94 to −0.8)
East Asia	8,326.79 (4,111.25 to 13,251.31)	10,234.93 (4,564.15 to 16,881.25)	905.7 (424.75 to 1,470.74)	480.97 (211.31 to 801.68)	−2.22 (−2.35 to −2.09)
Eastern Europe	585.05 (56.39 to 1,569.7)	530.72 (51.84 to 1,396.54)	207.86 (19.69 to 565.02)	159.76 (16.04 to 418.9)	−1.71 (−2.3 to −1.12)
Eastern Sub-Saharan Africa	329.83 (24.35 to 841.63)	363.83 (17.6 to 1,029.38)	422.32 (32.07 to 1,067.14)	230.64 (11.2 to 642.78)	−2.31 (−2.41 to −2.2)
High-income Asia Pacific	618.49 (191.07 to 1,110.42)	329.14 (50.39 to 724.35)	308.51 (93.76 to 556.95)	78.96 (12.3 to 172.24)	−5.33 (−5.58 to −5.09)
High-income North America	128.43 (8.16 to 436.69)	213.76 (14.18 to 623.08)	37.23 (2.32 to 124.58)	36.7 (2.48 to 105.33)	−0.04 (−0.17 to 0.1)
North Africa and Middle East	86.81 (12.94 to 349.81)	155.49 (24.3 to 605.7)	47.24 (7.81 to 192.69)	33.62 (5.78 to 133.26)	−1.22 (−1.25 to −1.19)
Oceania	7.63 (0.98 to 20.85)	17.67 (1.53 to 48.98)	281.03 (35.85 to 718.16)	264.15 (23.93 to 679.76)	−0.4 (−0.53 to −0.27)
South Asia	874.41 (63.12 to 2,523.07)	1,704.15 (135.2 to 4,602.3)	145.34 (9.97 to 425.24)	115.28 (9.02 to 318.31)	−0.64 (−0.75 to −0.54)
Southeast Asia	1,617.83 (508.03 to 2,951.83)	2,373.73 (413.92 to 4,877.25)	608.88 (187.95 to 1,107.36)	379.64 (66.34 to 783.15)	−1.65 (−1.74 to −1.56)
Southern Latin America	66.78 (3.97 to 178.33)	53.31 (2.77 to 145.32)	144.84 (8.56 to 386.97)	65.05 (3.4 to 177.16)	−3.06 (−3.26 to −2.87)
Southern Sub-Saharan Africa	32.6 (1.31 to 114.94)	40.04 (2.28 to 158.43)	105.85 (4.6 to 381.35)	67.75 (4.21 to 270.98)	−1.57 (−1.94 to −1.2)
Tropical Latin America	209.57 (11.45 to 555.61)	206.58 (12.74 to 549.03)	219.31 (12.05 to 578.82)	84.5 (5.24 to 224.83)	−3.53 (−3.64 to −3.42)
Western Europe	331.23 (26.2 to 1,041.44)	211.57 (16.49 to 658.83)	58.65 (4.78 to 181.34)	24.69 (1.91 to 75.23)	−3.21 (−3.44 to −2.99)
Western Sub-Saharan Africa	119.37 (4.75 to 462.69)	223.65 (8.43 to 821.67)	131.16 (5.41 to 504.93)	113.25 (4.42 to 415.22)	−0.42 (−0.47 to −0.36)

ASDR, age-standard DALYs rate; DALYs, disability-adjusted life years; EAPC, estimated annual percentage change.

Relentlessly declining trends were found in the EAPC of the ASDR of stroke attributable to DHIS from 1990 to 2019. During this time, there was a consistent downward trend observed globally for both sexes, with an estimated rate of change of −1.92 (95% CI: −2.02 to −1.82), which was more pronounced in females (−2.45; 95% CI: −2.57 to −2.34) (Table 2). This observation is consistent with the shifting trend observed in ASMR. While a general decreasing trend in ASDR was observed globally, our results at the regional level revealed that Middle SDI, Low-middle SDI, and Low SDI regions exhibited significantly higher EAPC values compared to High SDI and High-middle SDI regions. The region experiencing the most significant decrease was High-income Asia Pacific (−5.33; 95% CI: −5.58 to −5.09). Not surprisingly, the majority of these regions fall within the High and High-middle SDI categories. In contrast, High-income North America (−0.04; 95% CI: −0.17 to 0.1), classified as a High SDI region, demonstrated the smallest decrease in ASDR over the same period (Table 2). At the national level, we found that in twenty nations, the ASDR of stroke attributable to DHIS was on the rise. These nations were mostly found in Eastern Asia and other areas with lower SDIs. Pakistan has the highest EAPC value (1.47; 95% CI: 1.22 to 1.72) out of these nations. In contrast, the countries with the highest decrease in ASDR were Republic of Korea, with an EAPC value of −6.45 (95% CI: −6.73 to −6.17) (Figure 2B, Supplementary Table 2S).

### 3.2 Correlation between the burden of stroke attributable to DHIS with SDI

**Figure 3:**
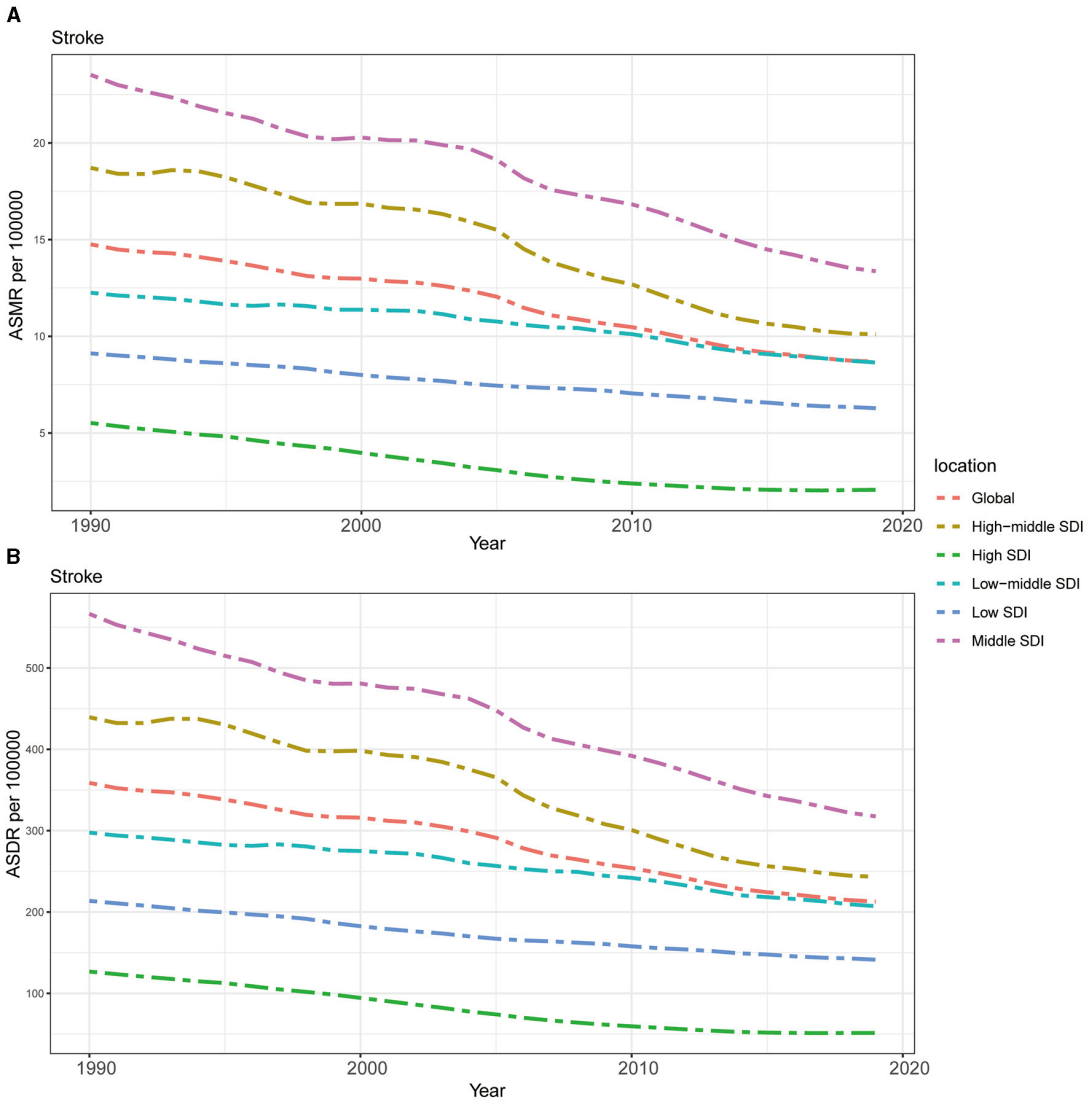
The burden of stroke attributable to diet high in sodium by SDI. **(A)** The ASMR and **(B)** ASDR of stroke attributable to diet high in sodium in different SDI regions from 1990 to 2019. Results are showed for both sexes worldwide. ASMR, age standardized mortality rate; DALYs, disease adjusted life year; ASDR, age standardized DALYs rate; SDI, sociodemographic index.

The ASMR and ASDR of stroke attributable to DHIS showed a steady decline from 1990 to 2019, both globally and in every GBD region, as shown in Figure 3. Notably, compared to the global average, the burden of ASMR and ASDR was higher in regions with moderate SDI levels, such as those designated as Middle SDI and High-middle SDI regions. On the other hand, regions with lower SDI values had a mortality burden that was relatively lower, with ASMR and ASDR being lower than the global average. The burden in High SDI regions was the lowest for both ASDR and ASMR among all GBD regions and much lower than the worldwide average, it is crucial to note, even while regions with moderate SDI levels carried a larger load than those with lower SDI values. Moreover, the decreasing speeds of the burden of stroke attributable to DHIS in Middle SDI and High-middle SDI regions were higher than other regions (Figure 3).

**Figure 4:**
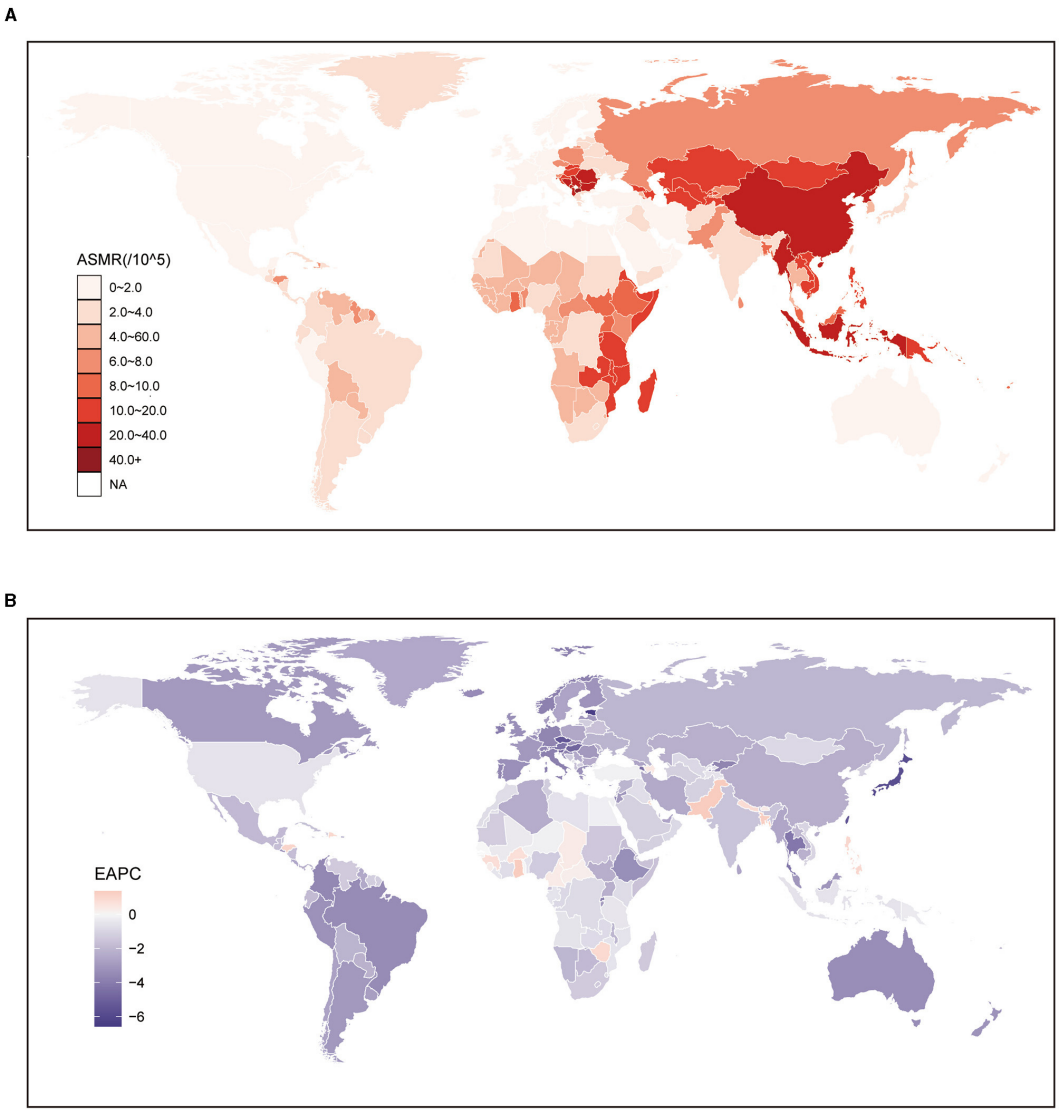
Correlations of ASMR as well as ASDR of stroke attributable to diet high in sodium and SDI at the regional level. The ASMR **(A)** and ASDR **(B)** of stroke attributable to diet high in sodium and SDI at the regional level in 21 regions from 1990 to 2019. ASMR, age standardized mortality rate; DALYs, disease adjusted life year; ASDR, age standardized DALYs rate.

To find out how the ASMR and ASDR of stroke attributable to DHIS relate to the SDI on a regional and national level, we performed correlation analysis. According to our research, regions with middle SDI values often had a higher stroke burden attributable to DHIS than other regions. Nevertheless, no meaningful correlations were discovered between ASMR and ASDR or SDI and ASMR (Figure 4). Based on their SDI values for the investigated period, Central Europe, East Asia, Southeast Asia, and Oceania showed higher ASMR than predicted at the regional level. On the other hand, considering their SDI levels, Australia, Southern Latin America, and North Africa and the Middle East saw lower ASMR rates than expected (Figure 4A). The ASDR in these regions likewise showed the same tendency (Figure 4B). Our study's conclusions, which contrasted the observed ASMR and ASDR with the levels predicted by national SDI values, agreed with regional findings. According to our data, North Macedonia had the greatest rates of ASMR and ASDR in 2019, but it also displayed the most differences in ASMR and ASDR for stroke that were attributable to DHIS (Supplementary Figure S1).

### 3.3 Age and sex patterns

**Figure 5:**
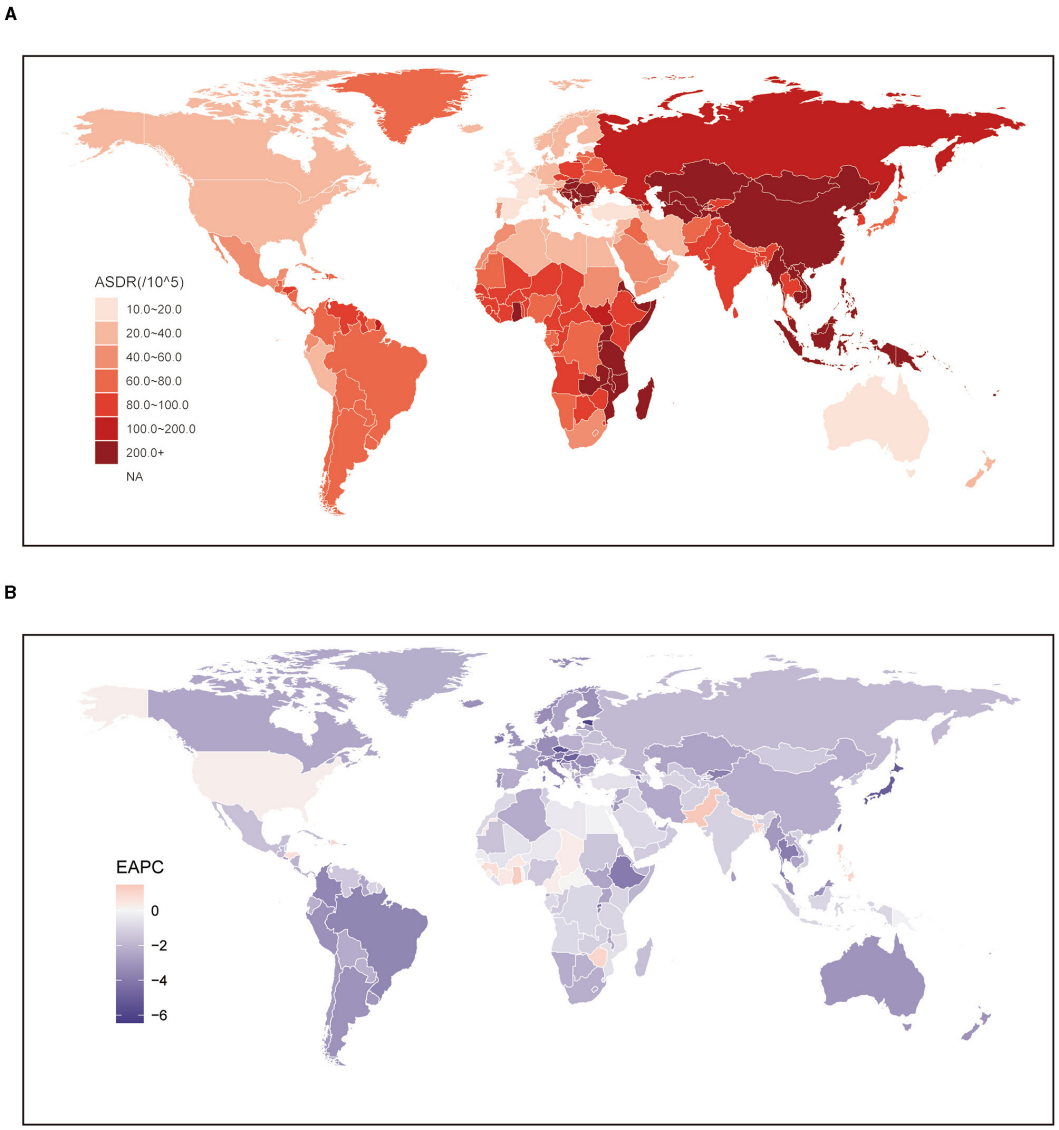
The burden of stroke attributable to diet high in sodium by age and sex. The all-cause ASMR **(A)** and ASDR **(B)** of stroke attributable to diet high in sodium worldwide in different age groups. ASMR, age standardized mortality rate; DALYs, disease adjusted life year; ASDR, age standardized DALYs rate.

In 2019, males displayed higher ASMR per 100,000 across all age groups. Moreover, this discrepancy amplified with age among individuals aged 25 to 94 years, with the most significant difference observed in the 90–94 age group (Figure 5A). Our research also revealed a predominant impact of ASDR of stroke attributed to DHIS among males. However, the most significant disparity was noted among individuals aged 75–79 years, with this difference diminishing among those older than 79 years (Figure 5B).

**Figure 6:**
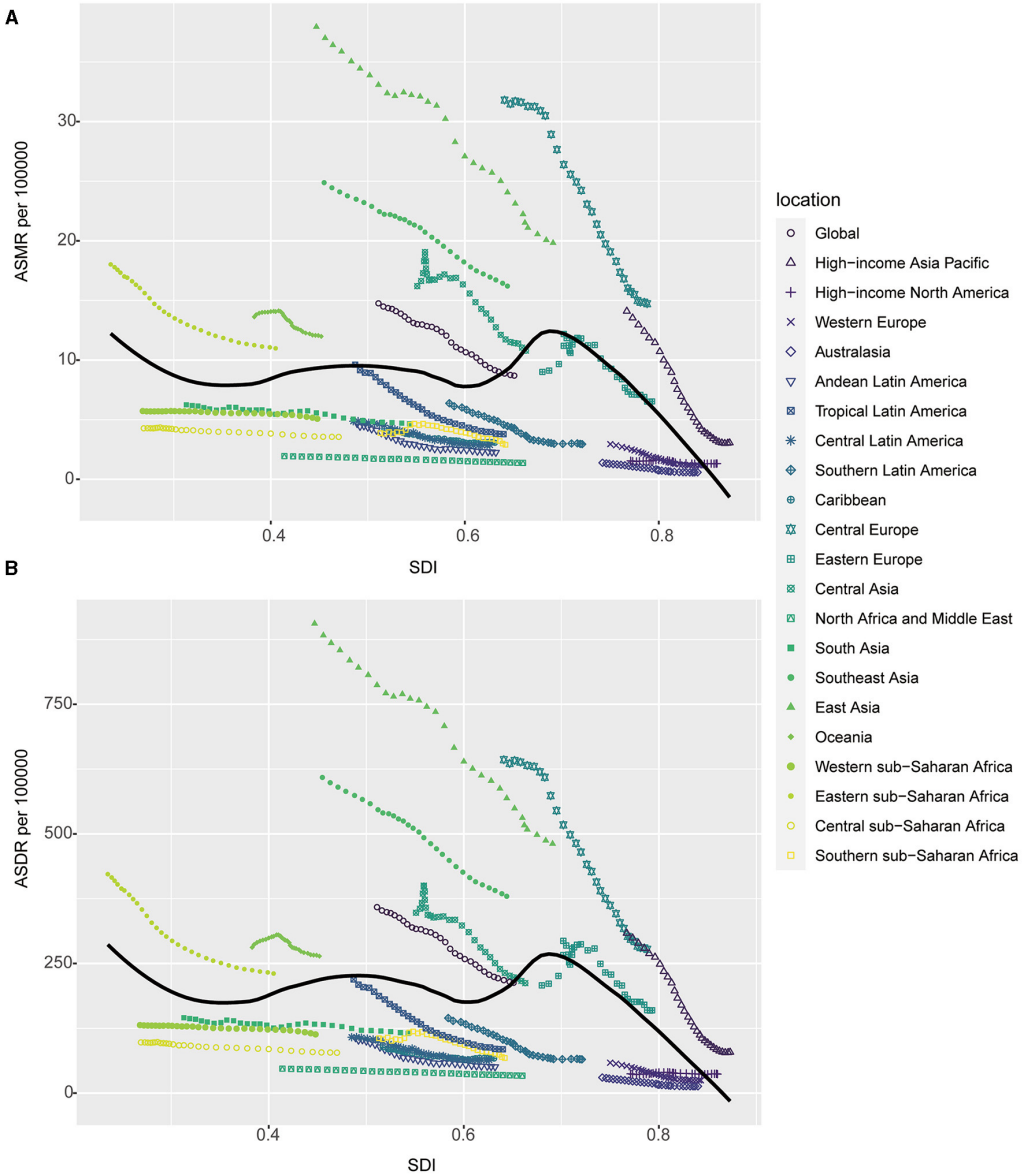
Global attributable burden of stroke attributable to diet high in sodium by sex. The age-standard ASMR **(A)** and ASDR **(B)** of stroke attributable to diet high in sodium by sex from 1990 to 2019. ASMR, age standardized mortality rate; DALYs, disease adjusted life year; ASDR, age standardized DALYs rate.

According to ASMR and ASDR, males continuously experienced a higher burden of stroke attributable to DHIS between 1990 and 2019. Moreover, these differences have not changed over time (Figure 6). Throughout this time, the rates of DALYs and the male-to-female mortality ratio both showed a general downward trend (Supplementary Figure S2). The ratio of male to female DALYs rate climbed continuously until age 39, at which point it reduced (Supplementary Figure S2). In contrast, the ratio of male to female mortality increased until age 34 and then fell. One interesting finding is that, in contrast to the general trend of change, the ratio of male to female mortality and the rate of DALYs both increased among those aged 50 to 64 and 84 to 89.

## 4 Discussion

Globally, among Level-3 causes, stroke continued to rank as the second most common cause of death and the third most common cause of death and disability overall in 2019. Furthermore, the absolute number of death cases and DALYs attributed to stroke continued to increase substantially from 1990 to 2019 (5). The huge increase in the worldwide burden of stroke was probably caused by a number of important risk factors being exposed to more people, in addition to population growth and aging. These include high body mass index, ambient particulate matter pollution, high fasting plasma glucose, elevated systolic blood pressure, alcohol consumption, low physical activity, kidney dysfunction, and DHIS (16, 17). We thoroughly analyzed the data in this study to determine the burden of stroke attributable to DHIS and to evaluate its correlation with age, year, and SDI. Overall, from 1990 to 2019, our research showed a considerable decline in the burden of stroke attributable to DHIS, especially for females. In comparison to other GBD regions, regions with moderate SDI values had a higher burden of stroke attributable to DHIS when the burden was examined at the regional level. While the burden in High SDI regions was not only the lowest in 2019, but the EAPC value of both ASMR and ASDR was also the lowest in these regions. This suggests a continuous decrease in burden from 1990 to 2019. It's crucial to recall that different regions saw different rates of decline, and there was no conclusive correlation between SDI and the DHIS-related stroke burden and EAPC values. In fact, during the previous three decades, the burden has significantly and quickly diminished. In the region, this tendency was also noted at the national level. Additionally, a negative link was found between SDI value and both metrics after looking at the relationship between ASMR and ASDR with SDI on a national scale. Consistent with our study's findings, these results imply that nations with higher SDI values typically have a lower stroke burden attributable to DHIS. Furthermore, compared to males, females generally showed lower ASMR and ASDR, according to our results. The age range of 30–34 was the peak for the gender disparity in ASDR, whereas the age range of 35–39 was the peak for ASMR.

For the first time, we have provided a comprehensive report on the global, regional, and national burden of stroke attributable to DHIS across all pathological types. Although ischemic stroke continues to account for the largest proportion of all new strokes (62.4%), followed by intracerebral hemorrhage (27.9%), and subarachnoid hemorrhage (9.7%) (2, 5). Our research demonstrated that the burden of stroke attributable to DHIS was higher in Middle and High-middle SDI regions among all GBD regions, consistent with previous published articles (5). Furthermore, it has been reported that the relative proportions of each pathological type varied significantly according to income levels. In Middle and High-middle SDI regions, a new stroke was nearly twice as likely to be intracerebral hemorrhage, whereas in High SDI regions, it was over twice as likely to be subarachnoid hemorrhage (5). The heightened risk of intracerebral hemorrhage in Middle and High-middle SDI regions may be linked to the significant clinical importance and population-attributable risk of hypertension, stemming from lower levels of awareness and control of hypertension in these areas (18). However, the specific pathological type of stroke was not addressed in our research, making it impossible to determine the relationship between the severe burden of stroke attributable to DHIS and the high prevalence of intracerebral hemorrhage in these regions. Further research might contribute to determining the specific pathological types of strokes attributable to DHIS at different levels, providing precise recommendations for policymakers in various regions.

Advancements in public awareness of stroke and the utilization of emergency medical services, improvements in medical treatment, as well as stroke risk factor prevention, collectively contribute to the overall declines in ASMR and ASDR of strokes attributable to DHIS. This is particularly pronounced in High SDI regions, aligning with the broader trend of strokes caused by all risk factors (4, 5, 12). However, the majority of the global burden of stroke persists in Middle and High-middle SDI regions. The proportion of DALYs was notably high in these areas, suggesting inadequate control and promotion of sodium intake. Similar with previous articles (5, 12–15), we also observed that the ASMR and ASDR of strokes attributable to DHIS were higher among males compared to females. These findings imply a potentially heightened risk of disability and death from stroke in males, contrasted with potentially improved survival rates among females. Gonadal hormone exposure plays a significant role in post-stroke outcomes, particularly regarding sex differences. Estrogen acts as a protective factor against stroke for women, especially before menopause (19). Additionally, women exhibited a lower incidence of hypertension and adopted healthier lifestyles compared to men (20), which could account for the lower stroke mortality and greater decline observed in women over the past 30 years. Aging stands as the most potent non-modifiable risk factor for stroke. Previous studies have shown that elderly adults experience a higher incidence of stroke and poorer functional recovery compared to younger individuals (21), leading to elevated mortality rates within the elderly population. Our research findings also indicated that the ASMR and ASDR of strokes attributed to DHIS were significantly elevated among the elderly population. This observation may be linked to an age-related increase in blood pressure salt sensitivity, which is associated with a gradual loss of functional nephrons (22). Another potential factor is that the combination of advanced age and preexisting comorbidities significantly increases the risk of stroke mortality (23).

Sodium is indeed an essential nutrient vital for normal bodily functions and overall health. Consequently, it is expected to maintain a physiologically “healthy” range of intake, akin to other essential electrolytes (24, 25). Several health organizations advocate for low sodium intake, typically below 2.3 grams per day (~1 teaspoon of salt), for the general population (6, 26). However, the majority of global populations consume between 3 to 6 grams of sodium daily. This indicates that the current recommended levels of below 2.3 grams per day for entire populations are significantly lower than what most people worldwide typically consume (27). The current evidence from cohort studies indicates a J-shaped relationship between sodium intake and cardiovascular events, which aligns with expectations for an essential nutrient (25, 28). High sodium intake has been identified as a risk factor for numerous non-communicable diseases, including hypertension, stomach cancer, renal diseases, and obesity (29). On one hand, sodium loading can result in an expansion of extracellular volume, leading to hypertension in cases where renal function has been compromised (30, 31). On the flip side, a compromised response of the Renin-Angiotensin-Aldosterone System during sodium manipulation could be associated with a blood pressure sensitivity to salt. This assumption stems from the belief that angiotensin II type 1 receptors might play a role in the elevation of blood pressure triggered by sodium intake (32).

While DHIS poses risks to various organs and overall human health, there are also potential dangers associated with significant sodium restriction in the diet. One such risk is iodine deficiency, which is prevalent in Alpine countries like Austria (33). The iodization of salt stands as a crucial and cost-effective strategy in combating iodine deficiency and its associated condition, hypothyroidism, among the general population. Decreasing salt consumption among the populace could exacerbate iodine deficiency, particularly in individuals with suboptimal or marginal iodine intake. Furthermore, in individuals undergoing acute dehydration or diarrhea, a low-salt diet may raise the risk of volume depletion and hypotension. Additionally, in elderly individuals, reducing dietary salt could lead to altered taste perceptions, which might subsequently increase the risk of low energy intake and malnutrition (34). So, while it is recommended to maintain sodium intake below 2.3 g/day, current recommendations to drastically reduce sodium intake across entire populations to low levels are premature. According to a recent review, the author suggests that employing targeted approaches aimed at individuals consuming high amounts of sodium (>5 g/day) would be a more suitable strategy. For at-risk individuals, particularly the elderly and those with hypertension, it's sensible to recommend avoiding excessive sodium intake (>4 g/day), especially in the absence of orthostatic intolerance syndromes (35).

Policymakers are paying more attention to the economic burden of continuous expansion brought on by aggravated medical requirements as a result of the population's recent rapid growth and aging. A previous study found that reducing salt intake from 9.5 to 8.1 g/d between 2003 and 2011 reduced stroke mortality in England by 42% (36). Research from China further supported the idea that cutting back on salt is an economical way to lessen the prevalence of cardiovascular disease thus decreasing the economic burden (37). While this study represented the first and most extensive review of the global, regional, and national burden of stroke attributable to DHIS across all three pathological types, it was not without limitations commonly found in previous GBD studies (2, 4, 5, 16). First and foremost, it's critical to acknowledge that there were gaps in the data sources' accessibility and thoroughness. Moreover, the lack of reputable medical resources in several developing nations further restricted the availability of primary data of excellent quality. Thirdly, the difficulty in separating mortality directly attributable to stroke from deaths attributable to its comorbidities suggests that the number of deaths from stroke may be underestimated. Last but not least, it's important to note that GBD 2019 did not incorporate the results of several ongoing national food surveys, including the Family Budget Survey and others, which may have provided further insightful information.

## 5 Conclusion

Despite the significantly increased absolute number of deaths and DALYs attributed to stroke caused by DHIS in the past three decades, there has been a notable decline in the ASMR and ASDR during this time, especially in regions with high SDI. The results at the national level were in line with those at the regional level, with regions with moderate SDI bearing the brunt of the load relative to other regions. Males showed consistently higher values than females in both ASMR and ASDR for stroke related to DHIS over the course of the thirty-year period. These results highlight the significance of giving extra nutrition education a high priority, with a focus on males worldwide. This initiative seeks to reduce the number of stroke cases attributable to DHIS and stop its rise, especially in regions and nations with moderate SDI values.

## Data Availability

The original contributions presented in the study are included in the article/Supplementary material, further inquiries can be directed to the corresponding author.

## Supplementary material

Supplementary Figure S1 Correlations of ASMR as well as ASDR and SDI at the national level. The ASMR **(A)** as well as ASDR **(B)** of stroke attributable to diet high in sodium and SDI at the regional level in 204 countries and territories from 1990 to 2019. ASMR, age standardized mortality rate; DALYs, disease adjusted life year; ASDR, age standardized DALYs rate; SDI, sociodemographic index.

Supplementary Figure S2 Sex disparity in the global exposure and attributable burden of stroke attributable to diet high in sodium in different years. Ratio of male to female ASMR **(A)** and ASDR **(B)** of stroke attributable to diet high in sodium from 1990 to 2019. ASMR, age standardized mortality rate; DALYs, disease adjusted life year; ASDR, age standardized DALYs rate.

Supplementary Table S1 Death number and age-standardized morality of Stroke attributable to Diet high in sodium for both sexes combined in 1990 and 2019, and EAPC of ASMR from 1990 to 2019 in 204 countries and territories.

Supplementary Table S2 DALYs number and age-standardized DALYs rate of Stroke attributable to Diet high in sodium for both sexes combined in 1990 and 2019, and EAPC of ASMR from 1990 to 2019 in 204 countries and territories.

## References

[1] Feigin V Norrving B Sudlow CLM Sacco RL. Updated criteria for population-based stroke and transient ischemic attack incidence studies for the 21st Century. Stroke. (2018) 49:2248–55. 10.1161/STROKEAHA.118.022161 30355005

[2] Collaborators GDaI. Global burden of 369 diseases and injuries in 204 countries and territories, 1990-2019: a systematic analysis for the Global Burden of Disease Study 2019. Lancet. (2020) 396:1204–22. 10.1016/S0140-6736(20)30925-9 33069326 PMC7567026

[3] Collaborators GDaH. Global, regional, and national disability-adjusted life-years (DALYs) for 359 diseases and injuries and healthy life expectancy (HALE) for 195 countries and territories, 1990-2017: a systematic analysis for the Global Burden of Disease Study 2017. Lancet. (2018) 392:1859–922. 10.1016/S0140-6736(18)32335-3 30415748 PMC6252083

[4] Saini V Guada L Yavagal DR. Global epidemiology of stroke and access to acute ischemic stroke interventions. Neurology. (2021) 97:S6–s16. 10.1212/WNL.0000000000012781 34785599

[5] Collaborators GS. Global, regional, and national burden of stroke and its risk factors, 1990-2019: a systematic analysis for the Global Burden of Disease Study 2019. Lancet Neurol. (2021) 20:795–820. 10.1016/S1474-4422(21)00252-0 34487721 PMC8443449

[6] WHO Guidelines Approved by the Guidelines Review Committee. Guideline: Sodium Intake for Adults and Children. Geneva: World Health Organization (2012). 23658998

[7] He FJ MacGregor GA. Reducing population salt intake worldwide: from evidence to implementation. Prog Cardiovasc Dis. (2010) 52:363–82. 10.1016/j.pcad.2009.12.006 20226955

[8] Filippini T Malavolti M Whelton PK Naska A Orsini N Vinceti M. Blood pressure effects of sodium reduction: dose-response meta-analysis of experimental studies. Circulation. (2021) 143:1542–67. 10.1161/CIRCULATIONAHA.120.050371 33586450 PMC8055199

[9] He FJ Li J Macgregor GA. Effect of longer term modest salt reduction on blood pressure: cochrane systematic review and meta-analysis of randomised trials. BMJ (Clinical research ed). (2013) 346:f1325. 10.1136/bmj.f1325 23558162

[10] Graudal N Hubeck-Graudal T Jürgens G Taylor RS. Dose-response relation between dietary sodium and blood pressure: a meta-regression analysis of 133 randomized controlled trials. Am J Clin Nutr. (2019) 109:1273–8. 10.1093/ajcn/nqy384 31051506

[11] Gupta DK Lewis CE Varady KA Su YR Madhur MS Lackland DT. Effect of dietary sodium on blood pressure: a crossover trial. Jama. (2023) 330:2258–66. 10.1001/jama.2023.23651 37950918 PMC10640704

[12] Ma Q Li R Wang L Yin P Wang Y Yan C. Temporal trend and attributable risk factors of stroke burden in China, 1990-2019: an analysis for the Global Burden of Disease Study 2019. The Lancet Public health. (2021) 6:e897–906. 10.1016/S2468-2667(21)00228-0 34838196 PMC9047702

[13] Ding Q Liu S Yao Y Liu H Cai T Han L. Global, regional, and national burden of ischemic stroke, 1990-2019. Neurology. (2022) 98:e279–e90. 10.1212/WNL.0000000000013115 34911748

[14] Cao J Eshak ES Liu K Gero K Liu Z Yu C. Age-period-cohort analysis of stroke mortality attributable to high sodium intake in China and Japan. Stroke. (2019) 50:1648–54. 10.1161/STROKEAHA.118.024617 31195942 PMC6594775

[15] Wang Y Wang J Chen S Li B Lu X Li J. Different changing patterns for stroke subtype mortality attributable to high sodium intake in China during 1990 to 2019. Stroke. (2023) 54:1078–87. 10.1161/STROKEAHA.122.040848 36727509

[16] Collaborators GRF. Global burden of 87 risk factors in 204 countries and territories, 1990-2019: a systematic analysis for the Global Burden of Disease Study 2019. Lancet. (2020) 396:1223–49. 10.1016/S0140-6736(20)30752-2 33069327 PMC7566194

[17] Collaborators. GV. Five insights from the Global Burden of Disease Study 2019. Lancet. (2020) 396:1135–59. 10.1016/S0140-6736(20)31404-5 33069324 PMC7116361

[18] Owolabi MO Sarfo F Akinyemi R Gebregziabher M Akpa O Akpalu A. Dominant modifiable risk factors for stroke in Ghana and Nigeria (SIREN): a case-control study. Lancet Global health. (2018) 6:e436–e46. 10.1016/S2214-109X(18)30002-0 29496511 PMC5906101

[19] Noh B McCullough LD Moruno-Manchon JF. Sex-biased autophagy as a potential mechanism mediating sex differences in ischemic stroke outcome. Neural Regener Res. (2023) 18:31–7. 10.4103/1673-5374.340406 35799505 PMC9241419

[20] Gillis EE Sullivan JC. Sex differences in hypertension: recent advances. Hypertension. (2016) 68:1322–7. 10.1161/HYPERTENSIONAHA.116.06602 27777357 PMC5159215

[21] Roy-O'Reilly M McCullough LD. Age and sex are critical factors in ischemic stroke pathology. Endocrinology. (2018) 159:3120–31. 10.1210/en.2018-00465 30010821 PMC6963709

[22] Hall JE. Renal dysfunction, rather than nonrenal vascular dysfunction, mediates salt-induced hypertension. Circulation. (2016) 133:894–906. 10.1161/CIRCULATIONAHA.115.018526 26927007 PMC5009905

[23] Lioutas VA Beiser AS Aparicio HJ Himali JJ Selim MH Romero JR. Assessment of incidence and risk factors of intracerebral hemorrhage among participants in the framingham heart study between 1948 and 2016. JAMA Neurol. (2020) 77:1252–60. 10.1001/jamaneurol.2020.1512 32511690 PMC7281354

[24] Weisman NW. The neuroscience of drives for food, water, and salt. N Engl J Med. (2019) 380:e33. 10.1056/NEJMc1902946 31042848

[25] Heaney RP. Sodium: how and how not to set a nutrient intake recommendation. Am J Hypertens. (2013) 26:1194–7. 10.1093/ajh/hpt130 24042548

[26] Whelton PK Carey RM Aronow WS Casey DE Jr., Collins KJ, Dennison Himmelfarb C, et al. 2017 ACC/AHA/AAPA/ABC/ACPM/AGS/APhA/ASH/ASPC/NMA/PCNA Guideline for the prevention, detection, evaluation, and management of high blood pressure in adults: executive summary: a report of the American College of Cardiology/American Heart Association task force on clinical practice guidelines. Hypertension. (2018) 71:1269–324. 10.1161/HYP.0000000000000066 29133354

[27] Micha R Khatibzadeh S Shi P Andrews KG Engell RE Mozaffarian D. Global, regional and national consumption of major food groups in 1990 and 2010: a systematic analysis including 266 country-specific nutrition surveys worldwide. BMJ Open. (2015) 5:e008705. 10.1136/bmjopen-2015-008705 26408285 PMC4593162

[28] Heaney RP. The nutrient problem, as seen through the lens of calcium. J Clin Endocrinol Metab. (2011) 96:2035–7. 10.1210/jc.2011-1545 21734005

[29] Rust P Ekmekcioglu C. Impact of salt intake on the pathogenesis and treatment of hypertension. Adv Exp Med Biol. (2017) 956:61–84. 10.1007/5584_2016_147 27757935

[30] Farquhar WB Edwards DG Jurkovitz CT Weintraub WS. Dietary sodium and health: more than just blood pressure. J Am Coll Cardiol. (2015) 65:1042–50. 10.1016/j.jacc.2014.12.039 25766952 PMC5098396

[31] Koomans HA Roos JC Boer P Geyskes GG Mees EJ. Salt sensitivity of blood pressure in chronic renal failure. Evidence for renal control of body fluid distribution in man. Hypertension. (1982) 4:190–7. 10.1161/01.HYP.4.2.190 7040224

[32] Crowley SD Gurley SB Oliverio MI Pazmino AK Griffiths R Flannery PJ. Distinct roles for the kidney and systemic tissues in blood pressure regulation by the renin-angiotensin system. J Clin Invest. (2005) 115:1092–9. 10.1172/JCI200523378 15841186 PMC1070417

[33] Burnier M Wuerzner G Bochud M. Salt, blood pressure and cardiovascular risk: what is the most adequate preventive strategy? A Swiss perspective. Front Physiol. (2015) 6:227. 10.3389/fphys.2015.00227 26321959 PMC4535281

[34] Zeanandin G Molato O Le Duff F Guérin O Hébuterne X Schneider SM. Impact of restrictive diets on the risk of undernutrition in a free-living elderly population. Clin Nutr. (2012) 31:69–73. 10.1016/j.clnu.2011.08.007 21872973

[35] Mente A O'Donnell M Yusuf S. Sodium intake and health: what should we recommend based on the current evidence? Nutrients. (2021) 13:3232. 10.3390/nu13093232 34579105 PMC8468043

[36] He FJ Pombo-Rodrigues S Macgregor GA. Salt reduction in England from 2003 to 2011: its relationship to blood pressure, stroke and ischaemic heart disease mortality. BMJ Open. (2014) 4:e004549. 10.1136/bmjopen-2013-004549 24732242 PMC3987732

[37] Neal B Wu Y Feng X Zhang R Zhang Y Shi J. Effect of salt substitution on cardiovascular events and death. N Engl J Med. (2021) 385:1067–77. 10.1056/NEJMoa2105675 34459569

