# Supplementary material for: Global, regional, and national burden of stroke attributable to diet high in sodium from 1990 to 2019: a systematic analysis from the global burden of disease study 2019
Source: Front Neurol. 2024 Aug 14;15:1437633. doi: 10.3389/fneur.2024.1437633 (PMC11349671; doi:10.3389/fneur.2024.1437633)
Supplement: Supplementary Table S1 — Death number and age-standardized morality of Stroke attributable to Diet high in sodium for both sexes combined in 1990 and 2019, and EAPC of ASMR from 1990 to 2019 in 204 countries and territories. [file Table_1.docx]

Table 1S. Death number and age-standardized morality of Stroke attributable to Diet high in sodium for both sexes combined in 1990 and 2019, and EAPC of ASMR from 1990 to 2019 in 204 countries and territories

| Location | Death number in 1990 | Death number in 2019 | ASMR in 1990 | ASMR in 2019 | EAPC 1990-2019 |
| --- | --- | --- | --- | --- | --- |
| Afghanistan | 230.34(27.38 to 988.22) | 307.27(37.98 to 1274.1) | 3.46(0.47 to 14.75) | 2.61(0.38 to 10.79) | -1.15 (-1.39 to -0.92) |
| Albania | 681.05(303.21 to 1103.68) | 1027.74(311.65 to 1949.53) | 39.48(17.15 to 65.28) | 24.52(7.4 to 46.92) | -1.69 (-1.86 to -1.51) |
| Algeria | 258.31(33.59 to 1076.93) | 395.08(63.4 to 1522.2) | 2.69(0.46 to 11.26) | 1.43(0.26 to 5.5) | -2.3 (-2.39 to -2.21) |
| American Samoa | 0.78(0.05 to 3.07) | 1.77(0.09 to 5.91) | 4.84(0.28 to 17.39) | 4.61(0.22 to 14.64) | -0.51 (-0.67 to -0.35) |
| Andorra | 0.49(0.03 to 1.83) | 1.02(0.07 to 3.82) | 1.09(0.08 to 4.2) | 0.66(0.05 to 2.46) | -1.73 (-1.94 to -1.51) |
| Angola | 183.86(9.13 to 793.92) | 439.87(18.68 to 1796.24) | 5.7(0.29 to 24.12) | 5.25(0.23 to 20.39) | -0.53 (-0.74 to -0.33) |
| Antigua and Barbuda | 2.77(0.13 to 8.9) | 2.64(0.14 to 8.93) | 4.89(0.23 to 15.86) | 2.92(0.15 to 9.84) | -2.21 (-2.47 to -1.94) |
| Argentina | 1899.41(104.04 to 5297.42) | 1550.73(75.43 to 4317.62) | 6.17(0.34 to 17.14) | 2.84(0.14 to 7.92) | -3.03 (-3.3 to -2.75) |
| Armenia | 273.47(50.06 to 595.77) | 175.87(14.28 to 467.5) | 12.19(2.26 to 26.78) | 4.43(0.37 to 11.68) | -4.39 (-4.75 to -4.02) |
| Australia | 224.2(25.51 to 912.9) | 232.05(26.74 to 964.47) | 1.21(0.14 to 4.89) | 0.51(0.06 to 2.07) | -3.36 (-3.53 to -3.19) |
| Austria | 578.12(29.95 to 1662.13) | 316.03(17.82 to 881.34) | 4.66(0.25 to 13.55) | 1.49(0.08 to 4.13) | -4.36 (-4.64 to -4.08) |
| Azerbaijan | 572.34(101.25 to 1276.36) | 719.63(51.9 to 1967.8) | 12.4(2.19 to 27.84) | 11.69(0.87 to 31.48) | 0.43 (0.04 to 0.83) |
| Bahamas | 5.37(0.25 to 18.01) | 9.22(0.45 to 31.68) | 3.94(0.19 to 13.03) | 2.59(0.13 to 8.86) | -1.7 (-1.86 to -1.53) |
| Bahrain | 2.34(0.28 to 9.44) | 5.08(0.6 to 20.39) | 1.61(0.26 to 6.52) | 0.76(0.14 to 3.13) | -2.62 (-2.9 to -2.34) |
| Bangladesh | 3059.1(146.26 to 10705.21) | 9576.23(460.07 to 27705.44) | 7.43(0.36 to 25.89) | 8.19(0.41 to 23.95) | 1.27 (0.67 to 1.88) |
| Barbados | 8.16(0.67 to 32.51) | 8.82(0.64 to 34.27) | 2.69(0.22 to 10.8) | 1.8(0.13 to 6.95) | -1.76 (-2.04 to -1.48) |
| Belarus | 518.72(37.33 to 1843.44) | 494.96(36.07 to 1839.73) | 4.08(0.3 to 14.5) | 3.09(0.22 to 11.47) | -1.66 (-2.14 to -1.17) |
| Belgium | 685.62(36.78 to 1918.84) | 478.75(22.35 to 1406.24) | 4.34(0.23 to 12.19) | 1.74(0.08 to 5.02) | -3.36 (-3.49 to -3.24) |
| Belize | 2.97(0.14 to 9.75) | 5.89(0.29 to 19.51) | 3.35(0.16 to 10.91) | 2.35(0.12 to 7.72) | -1.67 (-2.1 to -1.24) |
| Benin | 151.12(5.06 to 538.54) | 296.27(9.21 to 999.38) | 8.26(0.29 to 29.31) | 7.1(0.24 to 24.12) | -0.52 (-0.58 to -0.45) |
| Bermuda | 1.87(0.09 to 6.13) | 1.87(0.09 to 6.12) | 3.32(0.16 to 10.7) | 1.35(0.07 to 4.39) | -3.33 (-3.49 to -3.17) |
| Bhutan | 8.46(0.37 to 30.45) | 20.72(0.98 to 59.87) | 4.2(0.19 to 15.09) | 4.13(0.2 to 11.98) | 0.38 (0.17 to 0.59) |
| Bolivia (Plurinational State of) | 205.2(7.99 to 587.94) | 326.38(15.21 to 955.04) | 7.04(0.28 to 20.07) | 4.11(0.19 to 12.06) | -2.07 (-2.28 to -1.85) |
| Bosnia and Herzegovina | 989.61(407.41 to 1616.28) | 1214.81(374.48 to 2261.07) | 29.75(12.21 to 48.88) | 21.34(6.55 to 40.04) | -1.32 (-1.55 to -1.09) |
| Botswana | 35.94(1.49 to 137.25) | 53.9(2.54 to 228.25) | 6.82(0.33 to 26.49) | 4.42(0.25 to 19.03) | -1.87 (-2.18 to -1.56) |
| Brazil | 7791.81(441.68 to 20672.85) | 8663.75(566.3 to 22790.18) | 9.68(0.58 to 25.74) | 3.77(0.25 to 9.94) | -3.45 (-3.55 to -3.34) |
| Brunei Darussalam | 16.75(3.51 to 33.52) | 17.52(2.8 to 37.69) | 21.41(4.08 to 44.14) | 7.99(1.1 to 17.64) | -3.69 (-3.89 to -3.49) |
| Bulgaria | 5862.57(2556.45 to 9359.73) | 4672.63(1462.85 to 8576.8) | 52.26(22.93 to 84.32) | 30.91(9.83 to 56.76) | -2.34 (-2.53 to -2.15) |
| Burkina Faso | 164.09(6.04 to 652.43) | 383.44(13.49 to 1475.08) | 4.28(0.18 to 17.05) | 4.78(0.18 to 18.35) | 0.68 (0.58 to 0.79) |
| Burundi | 536.79(29.33 to 1405.2) | 431.4(12.63 to 1298.77) | 24.52(1.36 to 63.32) | 11.71(0.36 to 33.54) | -2.97 (-3.12 to -2.82) |
| Cabo Verde | 6.89(0.3 to 25.65) | 17.09(0.66 to 64.47) | 3.02(0.13 to 11.23) | 4.07(0.16 to 15.26) | 0.24 (-0.06 to 0.55) |
| Cambodia | 1115.44(298.51 to 2131.5) | 1660.5(202.68 to 3668.22) | 27.04(6.55 to 53.23) | 15.8(1.8 to 35.94) | -2.18 (-2.31 to -2.04) |
| Cameroon | 210.89(8.13 to 839.21) | 582.84(19.36 to 2270.9) | 5.36(0.23 to 21.58) | 5.62(0.21 to 21.76) | 0.34 (0.04 to 0.64) |
| Canada | 676.08(38.55 to 2068.88) | 775.11(46.99 to 2451.34) | 2.12(0.12 to 6.53) | 1.05(0.06 to 3.22) | -2.97 (-3.16 to -2.78) |
| Central African Republic | 66.95(3.44 to 300.4) | 126.46(4.95 to 544.74) | 6.74(0.35 to 29.36) | 7.27(0.29 to 30.85) | 0.23 (0.13 to 0.32) |
| Chad | 141.11(5.55 to 556.89) | 290.81(9.85 to 1103.82) | 5.47(0.23 to 21.3) | 5.84(0.22 to 21.8) | 0.37 (0.22 to 0.53) |
| Chile | 623.24(36.56 to 1592.45) | 731.28(34.6 to 1944.17) | 6.81(0.4 to 17.45) | 3.06(0.15 to 8.12) | -2.68 (-2.76 to -2.6) |
| China | 297331.16(135653.82 to 496308.89) | 386518.04(152773.81 to 673800.71) | 38.44(15.9 to 67.18) | 20.07(7.52 to 36.21) | -2.24 (-2.4 to -2.08) |
| Colombia | 1375.47(286.7 to 2849.33) | 1932.01(413.57 to 4129.91) | 8.67(1.77 to 18.17) | 3.56(0.76 to 7.67) | -3.57 (-3.76 to -3.39) |
| Comoros | 37.18(2 to 97.55) | 42.82(1.3 to 120.76) | 18.16(1.02 to 46.95) | 9.82(0.3 to 27.79) | -2.44 (-2.62 to -2.27) |
| Congo | 60.67(3.13 to 267.1) | 115.24(4.77 to 459.33) | 6.82(0.35 to 28.86) | 5.74(0.24 to 22.38) | -0.86 (-1.02 to -0.7) |
| Cook Islands | 1.12(0.14 to 2.65) | 1.38(0.11 to 3.22) | 10.16(1.26 to 23.93) | 5.55(0.42 to 13.15) | -2.4 (-2.61 to -2.19) |
| Costa Rica | 63.28(2.99 to 161.02) | 110.91(8.04 to 285.63) | 3.84(0.18 to 9.91) | 2.15(0.16 to 5.51) | -2.48 (-2.85 to -2.12) |
| Croatia | 1988.43(836.91 to 3237.87) | 1218.04(385.75 to 2289.99) | 33.6(14.11 to 55.19) | 12.76(4.07 to 23.88) | -3.7 (-3.88 to -3.51) |
| Cuba | 311.18(15.34 to 1006.41) | 428.89(22.67 to 1433.79) | 3.16(0.16 to 10.16) | 2.15(0.11 to 7.17) | -1.67 (-1.84 to -1.5) |
| Cyprus | 25.05(1.57 to 86.65) | 26.05(1.66 to 88.83) | 4.1(0.27 to 14.05) | 1.54(0.1 to 5.35) | -3.9 (-4.05 to -3.76) |
| Czechia | 4659.54(1976.02 to 7841.79) | 1749.14(543.59 to 3351.91) | 34.45(14.51 to 58.55) | 7.82(2.44 to 14.85) | -5.49 (-5.72 to -5.26) |
| Cte d'Ivoire | 234.14(7.36 to 850.38) | 510.29(15.64 to 1868.68) | 6.84(0.24 to 24.84) | 5.67(0.19 to 20.73) | -0.69 (-0.86 to -0.52) |
| Democratic People's Republic of Korea | 5755.15(2348.03 to 9889.23) | 8552.13(2310.31 to 16528.18) | 37.85(14.49 to 67.53) | 27.53(7.4 to 53.68) | -1.17 (-1.36 to -0.98) |
| Democratic Republic of the Congo | 462.5(36.42 to 2184.21) | 818.06(66.7 to 4126.75) | 3.45(0.3 to 15.95) | 2.71(0.24 to 13.38) | -0.84 (-0.91 to -0.77) |
| Denmark | 240.7(14.14 to 817.25) | 168.13(9.94 to 570.6) | 2.88(0.16 to 9.7) | 1.36(0.08 to 4.54) | -2.97 (-3.13 to -2.8) |
| Djibouti | 19.04(1.02 to 49.36) | 45.15(1.32 to 132.42) | 16.5(0.96 to 41.64) | 9.94(0.31 to 28.26) | -1.98 (-2.04 to -1.91) |
| Dominica | 2.94(0.14 to 9.42) | 2.7(0.14 to 9.36) | 3.97(0.19 to 12.83) | 2.96(0.15 to 10.26) | -0.85 (-1.02 to -0.69) |
| Dominican Republic | 118.08(5.6 to 391.02) | 347.58(16.53 to 1184.63) | 3.69(0.17 to 11.76) | 3.94(0.19 to 13.39) | 0.96 (0.73 to 1.19) |
| Ecuador | 224.65(9.02 to 604.8) | 362.3(17.54 to 1058.91) | 4.51(0.19 to 12.04) | 2.61(0.13 to 7.63) | -1.85 (-2.15 to -1.55) |
| Egypt | 473.64(56.8 to 1908.52) | 874.95(100.76 to 3640.8) | 1.7(0.24 to 6.87) | 1.47(0.2 to 6.11) | -0.21 (-0.34 to -0.08) |
| El Salvador | 158.19(8.51 to 408.37) | 157.76(10.48 to 423.11) | 5.44(0.29 to 14.21) | 2.54(0.17 to 6.84) | -2.97 (-3.43 to -2.5) |
| Equatorial Guinea | 10.37(0.52 to 46.56) | 15.6(0.63 to 61.89) | 6.1(0.3 to 27.25) | 4.25(0.18 to 16.48) | -1.69 (-1.89 to -1.48) |
| Eritrea | 174.03(9.57 to 456.93) | 261.67(8 to 783.25) | 19.63(1.13 to 51.9) | 12.5(0.39 to 36.63) | -1.76 (-1.83 to -1.68) |
| Estonia | 67.76(8.89 to 255.23) | 21.87(2.86 to 83.5) | 3.36(0.45 to 12.5) | 0.78(0.1 to 2.92) | -6.43 (-7 to -5.85) |
| Eswatini | 17.04(0.73 to 67.58) | 23.69(1.11 to 97.51) | 6.34(0.31 to 25.41) | 4.64(0.25 to 20.25) | -0.77 (-1.09 to -0.45) |
| Ethiopia | 3440.46(287.7 to 8828.95) | 2794.59(119.37 to 7988.15) | 19.24(1.65 to 48.72) | 8.17(0.35 to 23.42) | -3.33 (-3.49 to -3.18) |
| Fiji | 36.37(4.34 to 88.92) | 59.1(4.67 to 146.29) | 13.65(1.59 to 32.45) | 9.76(0.78 to 23.36) | -1.84 (-2.14 to -1.54) |
| Finland | 229.85(13.99 to 769.01) | 183.79(12.75 to 615.83) | 3.28(0.2 to 11.03) | 1.37(0.09 to 4.41) | -3.15 (-3.36 to -2.93) |
| France | 1727.93(117.37 to 6236.91) | 1437.35(94.46 to 5101.94) | 1.96(0.13 to 7.12) | 0.84(0.05 to 2.95) | -2.98 (-3.13 to -2.83) |
| Gabon | 24.38(1.33 to 103.53) | 37.95(1.63 to 150.98) | 5.05(0.28 to 20.99) | 4.48(0.2 to 17.3) | -0.57 (-0.78 to -0.36) |
| Gambia | 15.47(0.56 to 61.63) | 49.45(1.78 to 186.82) | 5.08(0.21 to 19.94) | 5.81(0.22 to 21.53) | 0.56 (0.42 to 0.7) |
| Georgia | 1325.14(251.74 to 2856.01) | 740.24(56.81 to 1935.83) | 24(4.61 to 51.44) | 11.38(0.88 to 29.85) | -2.58 (-2.91 to -2.24) |
| Germany | 4040.89(261.38 to 13562.86) | 2525.79(169.35 to 8455) | 3.12(0.2 to 10.49) | 1.19(0.08 to 3.89) | -3.69 (-4.06 to -3.31) |
| Ghana | 433.7(13.78 to 1591.83) | 1334.5(36.2 to 4234.03) | 8.14(0.29 to 30.05) | 9.68(0.29 to 30.76) | 1.33 (1.02 to 1.64) |
| Greece | 712.95(46.08 to 2528.69) | 667.5(46.18 to 2301.35) | 4.96(0.32 to 17.45) | 2.3(0.15 to 7.62) | -3.27 (-3.54 to -3) |
| Greenland | 1.87(0.1 to 6) | 2.19(0.12 to 6.66) | 6.43(0.39 to 20.9) | 3.57(0.21 to 10.96) | -2.48 (-2.69 to -2.27) |
| Grenada | 5.68(0.28 to 18.16) | 3.66(0.19 to 12.5) | 7.18(0.35 to 23.07) | 3.68(0.2 to 12.45) | -2.51 (-2.69 to -2.32) |
| Guam | 4.78(0.56 to 11.15) | 8.21(0.67 to 19.67) | 8.95(1.08 to 20.39) | 4.42(0.36 to 10.59) | -2.97 (-3.36 to -2.59) |
| Guatemala | 142.11(7.68 to 371.5) | 341.36(17.78 to 909.25) | 4.57(0.25 to 12.15) | 3.36(0.17 to 9.03) | -1.73 (-2.03 to -1.42) |
| Guinea | 160.8(6.36 to 641.36) | 294.85(10.56 to 1097.98) | 5.32(0.22 to 21.3) | 5.85(0.22 to 21.81) | 0.74 (0.61 to 0.88) |
| Guinea-Bissau | 29.28(1.02 to 115.24) | 47.86(1.58 to 177) | 7.78(0.3 to 31.09) | 7.41(0.27 to 27.52) | 0.08 (-0.02 to 0.19) |
| Guyana | 43.35(2.04 to 147.72) | 34.91(1.66 to 121.79) | 12.64(0.6 to 42.38) | 6.3(0.31 to 21.97) | -2.47 (-2.7 to -2.24) |
| Haiti | 264.46(11.54 to 957.4) | 403.14(17.16 to 1554.39) | 9.7(0.42 to 33.84) | 6.82(0.3 to 25.8) | -1.15 (-1.24 to -1.06) |
| Honduras | 119.16(6.28 to 308.73) | 434.3(30 to 1141.93) | 6.14(0.31 to 16.11) | 7.93(0.54 to 21.1) | 1.05 (0.85 to 1.24) |
| Hungary | 5595.83(2794.88 to 8527.98) | 2353.83(868.6 to 4146.49) | 39.3(19.58 to 60.45) | 11.59(4.27 to 20.48) | -4.81 (-5.05 to -4.58) |
| Iceland | 6.88(0.39 to 21.89) | 6.13(0.34 to 19.13) | 2.3(0.13 to 7.3) | 0.97(0.05 to 2.97) | -3.43 (-3.61 to -3.26) |
| India | 24764.48(1759.11 to 70833.18) | 44084.1(3612.49 to 125396.36) | 6.12(0.42 to 17.99) | 3.98(0.32 to 11.5) | -1.56 (-1.66 to -1.46) |
| Indonesia | 24793.12(7536.55 to 44735.54) | 43182.84(7737.27 to 89540.59) | 27.76(7.73 to 52.28) | 23.04(3.85 to 48.8) | -0.55 (-0.59 to -0.51) |
| Iran (Islamic Republic of) | 429.79(66.53 to 1630.98) | 684.77(136.36 to 2638.6) | 1.96(0.38 to 7.51) | 1.04(0.22 to 4.09) | -2.45 (-2.65 to -2.24) |
| Iraq | 223.53(28.46 to 927.98) | 534.59(68.52 to 2109.81) | 3.01(0.42 to 12.36) | 2.57(0.38 to 10.11) | -0.71 (-0.78 to -0.64) |
| Ireland | 89.24(7.04 to 333.63) | 61.24(5.17 to 234.05) | 2.24(0.18 to 8.41) | 0.79(0.07 to 3.01) | -3.99 (-4.24 to -3.73) |
| Israel | 110.45(6.48 to 370.88) | 124.98(7.7 to 415.33) | 2.4(0.14 to 7.98) | 1.01(0.06 to 3.29) | -3.6 (-3.85 to -3.34) |
| Italy | 3898.43(303.94 to 11184.24) | 2612.44(201.34 to 8227.4) | 4.46(0.35 to 12.67) | 1.54(0.12 to 4.55) | -3.87 (-4.15 to -3.59) |
| Jamaica | 88.98(4.49 to 299.69) | 112.94(5.89 to 384.33) | 4.87(0.25 to 16.52) | 3.59(0.18 to 12.23) | -1.13 (-1.48 to -0.77) |
| Japan | 19047.35(5787.59 to 35522.65) | 11558.5(1367.54 to 27752.46) | 11.98(3.51 to 22.56) | 2.62(0.32 to 6.01) | -5.8 (-6.06 to -5.53) |
| Jordan | 24.57(3.69 to 97.65) | 59.35(8.79 to 234.2) | 2.27(0.4 to 8.96) | 1.14(0.2 to 4.44) | -3.01 (-3.31 to -2.7) |
| Kazakhstan | 2038.78(361.1 to 4348.54) | 1629.07(119.16 to 4400.09) | 18.08(3.18 to 39.19) | 11.61(0.83 to 30.63) | -2.21 (-2.51 to -1.91) |
| Kenya | 576.9(54.95 to 1615.83) | 1099.94(102.37 to 3179.72) | 8.26(0.78 to 22.88) | 6.42(0.59 to 18.33) | -0.79 (-0.85 to -0.72) |
| Kiribati | 8(0.88 to 20.08) | 11.69(0.91 to 29.84) | 25.09(2.79 to 61.13) | 20.36(1.65 to 50.96) | -1.03 (-1.16 to -0.9) |
| Kuwait | 7.99(0.52 to 26.76) | 34.21(2.02 to 113.84) | 1.5(0.11 to 5.5) | 1.53(0.1 to 5.42) | 0.99 (0.26 to 1.72) |
| Kyrgyzstan | 504.02(89.89 to 1096.34) | 299.32(22.98 to 809.12) | 17.25(3.1 to 37.7) | 7.18(0.58 to 19.57) | -3.71 (-4.18 to -3.25) |
| Lao People's Democratic Republic | 605.9(162.5 to 1160.98) | 748.71(105.85 to 1633.53) | 30.85(7.71 to 61.73) | 19.21(2.48 to 42) | -1.97 (-2.13 to -1.8) |
| Latvia | 263.34(19.29 to 833.53) | 165.58(12.36 to 547.94) | 7.43(0.55 to 23.43) | 3.87(0.28 to 12.74) | -2.98 (-3.32 to -2.63) |
| Lebanon | 15.51(2.48 to 64.78) | 25.55(4.28 to 108.61) | 0.81(0.15 to 3.38) | 0.5(0.09 to 2.13) | -1.5 (-1.64 to -1.37) |
| Lesotho | 52.41(2.3 to 209.88) | 68.95(3.24 to 287.77) | 5.78(0.28 to 23.05) | 6.05(0.33 to 25.57) | 0.81 (0.54 to 1.08) |
| Liberia | 55.14(2.1 to 209.43) | 83.34(2.87 to 306.82) | 5.55(0.23 to 21.07) | 4.69(0.17 to 17.38) | -0.5 (-0.63 to -0.38) |
| Libya | 23.7(3.29 to 97.35) | 55.31(7.68 to 228.27) | 1.37(0.2 to 5.63) | 1.16(0.17 to 4.86) | -0.63 (-0.81 to -0.44) |
| Lithuania | 182.35(9.88 to 590.38) | 188.81(10.22 to 641.55) | 4.06(0.22 to 13.07) | 2.99(0.16 to 9.96) | -1.27 (-1.55 to -0.99) |
| Luxembourg | 21.01(1.35 to 71.83) | 12.4(0.79 to 41.53) | 3.96(0.25 to 13.44) | 1.12(0.07 to 3.69) | -4.53 (-4.65 to -4.42) |
| Madagascar | 1073.96(64.92 to 2723.89) | 1493.04(41.75 to 4457.42) | 22.49(1.43 to 56.06) | 16.5(0.46 to 47.5) | -1.38 (-1.56 to -1.21) |
| Malawi | 556.5(34.54 to 1397.49) | 625.24(19.21 to 1755.83) | 16.8(1.09 to 41.17) | 10.34(0.32 to 28.89) | -1.98 (-2.15 to -1.8) |
| Malaysia | 2068.43(557.25 to 3765.11) | 2429.25(312.35 to 5276.84) | 24.35(6.25 to 45.17) | 9.99(1.2 to 22.16) | -3.07 (-3.32 to -2.83) |
| Maldives | 17.8(5.39 to 32.2) | 17.13(2.15 to 37.53) | 22.29(6 to 42.72) | 6.63(0.82 to 14.74) | -4.93 (-5.26 to -4.6) |
| Mali | 208.45(7.98 to 820.6) | 378.63(13.86 to 1453.89) | 5.85(0.24 to 22.73) | 5.21(0.2 to 19.97) | -0.35 (-0.43 to -0.26) |
| Malta | 14.58(0.84 to 46.99) | 13.87(0.87 to 43.11) | 3.59(0.21 to 11.64) | 1.44(0.09 to 4.31) | -3.17 (-3.3 to -3.04) |
| Marshall Islands | 2.77(0.32 to 6.62) | 4.23(0.35 to 11.02) | 20.2(2.25 to 46.75) | 15.93(1.36 to 40.11) | -0.8 (-0.89 to -0.71) |
| Mauritania | 55.56(2.23 to 216.74) | 72.15(2.67 to 275.65) | 6.07(0.26 to 23.87) | 3.9(0.15 to 14.72) | -1.3 (-1.45 to -1.15) |
| Mauritius | 167.07(46.09 to 306.89) | 117.2(14.92 to 259.69) | 23.86(6.12 to 45.01) | 6.96(0.82 to 15.6) | -5.47 (-6.03 to -4.9) |
| Mexico | 1089.07(56.11 to 3264.35) | 1983(109.5 to 5848.09) | 2.94(0.16 to 8.84) | 1.8(0.1 to 5.33) | -1.88 (-2.02 to -1.74) |
| Micronesia (Federated States of) | 7.9(1.01 to 19.55) | 8.96(0.77 to 23.04) | 20.08(2.52 to 48.84) | 15.83(1.4 to 39.17) | -1.07 (-1.17 to -0.96) |
| Monaco | 2.16(0.13 to 7.83) | 1.39(0.09 to 5.17) | 2.71(0.16 to 9.66) | 1.24(0.07 to 4.48) | -2.76 (-3.18 to -2.33) |
| Mongolia | 198.43(35.48 to 453.03) | 331.68(26.39 to 921.81) | 20.65(3.7 to 46.56) | 16.72(1.32 to 45.33) | -0.87 (-1.37 to -0.36) |
| Montenegro | 249.14(109.22 to 419.34) | 332.52(107.29 to 615.42) | 43.83(19 to 74.39) | 35.97(11.51 to 66.71) | -0.65 (-0.8 to -0.5) |
| Morocco | 288.37(35.91 to 1194.49) | 529.66(72.52 to 2126.78) | 2.31(0.34 to 9.38) | 1.93(0.29 to 7.66) | -0.77 (-0.9 to -0.64) |
| Mozambique | 1095.21(72.84 to 2689.64) | 1526.16(43.93 to 4399.63) | 20.58(1.35 to 50.96) | 16.3(0.47 to 45.62) | -0.58 (-0.69 to -0.47) |
| Myanmar | 9572.02(2722.86 to 18200.91) | 9713.11(1250.27 to 20604.07) | 43.34(11.52 to 83.46) | 23.04(2.85 to 50.13) | -2.53 (-2.68 to -2.38) |
| Namibia | 43.94(1.99 to 171.42) | 52.38(2.91 to 217.14) | 6.68(0.33 to 26.1) | 4.1(0.25 to 17.17) | -2 (-2.25 to -1.74) |
| Nauru | 0.56(0.07 to 1.44) | 0.52(0.04 to 1.43) | 22.75(2.91 to 53.48) | 18.55(1.44 to 45.3) | -0.88 (-1.32 to -0.44) |
| Nepal | 355.18(15.77 to 1256.25) | 936.29(45.89 to 2738.05) | 4.39(0.21 to 15.86) | 4.72(0.24 to 13.81) | 0.83 (0.5 to 1.15) |
| Netherlands | 357.83(28.37 to 1351.45) | 396.33(27.05 to 1404.22) | 1.78(0.14 to 6.76) | 1.08(0.07 to 3.81) | -2.43 (-2.78 to -2.09) |
| New Zealand | 86.59(5.5 to 299.45) | 82.88(5.99 to 295.87) | 2.24(0.15 to 7.71) | 0.99(0.07 to 3.47) | -3.2 (-3.38 to -3.03) |
| Nicaragua | 79.53(4.03 to 201) | 150.68(9.39 to 386.02) | 5.68(0.28 to 14.38) | 3.96(0.25 to 10.34) | -1.75 (-2.03 to -1.47) |
| Niger | 146.13(5.26 to 576.31) | 364.26(12.1 to 1401.96) | 5.99(0.24 to 24.02) | 5.54(0.2 to 21.84) | -0.18 (-0.23 to -0.12) |
| Nigeria | 2094.03(96.55 to 8581.98) | 2823.05(128.51 to 10889) | 5.42(0.27 to 22.12) | 3.87(0.19 to 14.9) | -1.31 (-1.44 to -1.18) |
| Niue | 0.35(0.04 to 0.79) | 0.23(0.02 to 0.55) | 14.95(1.85 to 34.2) | 10.66(0.83 to 25.76) | -1.54 (-1.68 to -1.4) |
| North Macedonia | 937.35(409.01 to 1548.6) | 1166.06(367.87 to 2154.07) | 59.24(25.74 to 99.43) | 43.98(13.86 to 82.19) | -1.28 (-1.46 to -1.11) |
| Northern Mariana Islands | 1.56(0.18 to 3.99) | 3.89(0.35 to 9.05) | 14.75(1.7 to 34.72) | 9.21(0.82 to 21.65) | -1.92 (-2.13 to -1.7) |
| Norway | 183.47(15.31 to 635.77) | 99.99(8.72 to 343.17) | 2.51(0.2 to 8.5) | 0.92(0.08 to 3.11) | -3.81 (-3.93 to -3.69) |
| Oman | 14.04(1.63 to 59.64) | 19.95(2.52 to 80.02) | 2.38(0.35 to 10.23) | 1.56(0.26 to 6.62) | -0.9 (-1.13 to -0.67) |
| Pakistan | 2789.54(124.63 to 9796.56) | 7021.19(366.75 to 19292.87) | 5.37(0.25 to 18.46) | 7.3(0.4 to 20.18) | 1.36 (1.11 to 1.6) |
| Palau | 1.18(0.15 to 2.82) | 1.97(0.18 to 4.81) | 13.44(1.61 to 31.95) | 10.63(1.03 to 25.18) | -0.96 (-1.04 to -0.88) |
| Palestine | 20.87(3.09 to 82.39) | 33.45(5.23 to 134.04) | 2.66(0.44 to 10.62) | 1.76(0.31 to 6.92) | -1.48 (-1.57 to -1.39) |
| Panama | 80.55(4.49 to 203.54) | 136.62(9.57 to 348.52) | 5.67(0.31 to 14.3) | 3.24(0.23 to 8.26) | -2.21 (-2.35 to -2.07) |
| Papua New Guinea | 181.08(20.37 to 475.81) | 438.66(32.79 to 1167.35) | 12.96(1.44 to 32.4) | 12.01(0.94 to 30.22) | -0.33 (-0.42 to -0.24) |
| Paraguay | 147.01(5.94 to 397.86) | 216.97(10.41 to 622.36) | 7.05(0.28 to 19.29) | 4.08(0.2 to 11.69) | -2.14 (-2.29 to -1.99) |
| Peru | 464.66(19.8 to 1265.99) | 538.43(27.39 to 1557.77) | 4.14(0.18 to 11.32) | 1.65(0.08 to 4.77) | -3.28 (-3.63 to -2.93) |
| Philippines | 2697.09(749.18 to 5063.26) | 8858.63(1363.52 to 19148.95) | 11.03(2.7 to 21.81) | 12.14(1.83 to 26.48) | 0.94 (0.34 to 1.54) |
| Poland | 5128.97(1063.24 to 10657.48) | 4973.28(909.78 to 10522.83) | 12.7(2.73 to 26.41) | 6.8(1.24 to 14.37) | -2.5 (-2.61 to -2.4) |
| Portugal | 626.34(58.94 to 2412.21) | 470.95(41.44 to 1622.95) | 4.76(0.47 to 18.27) | 1.83(0.15 to 5.92) | -3.67 (-3.87 to -3.46) |
| Puerto Rico | 70.35(3.33 to 221.04) | 77.93(3.77 to 256.64) | 2.08(0.1 to 6.65) | 0.95(0.04 to 3.1) | -3.36 (-3.61 to -3.1) |
| Qatar | 1.36(0.14 to 5.38) | 4.69(0.42 to 18.33) | 1.29(0.2 to 5.38) | 0.78(0.13 to 3.14) | -1.64 (-1.88 to -1.4) |
| Republic of Korea | 6957.42(1409.93 to 13662.08) | 4615.18(948.01 to 9541.79) | 29.62(5.91 to 59.27) | 5.47(1.1 to 11.33) | -6.59 (-6.9 to -6.29) |
| Republic of Moldova | 218.63(14.68 to 772.66) | 179.78(12.34 to 657.04) | 5.45(0.39 to 19.05) | 3.1(0.21 to 11.21) | -2.22 (-2.55 to -1.89) |
| Romania | 10599.52(4627.76 to 17107.51) | 8853.03(2712.35 to 16052.84) | 42.41(17.9 to 70.35) | 22.23(6.86 to 40.18) | -3.01 (-3.32 to -2.7) |
| Russian Federation | 19280.83(1757.33 to 52119.64) | 18657.92(1672.57 to 50848.3) | 11.33(1.03 to 31.35) | 7.98(0.73 to 21.8) | -2.02 (-2.64 to -1.39) |
| Rwanda | 702.19(41.79 to 1784.07) | 515.66(15.16 to 1485.3) | 26.55(1.62 to 67.77) | 10.72(0.32 to 30.3) | -3.96 (-4.29 to -3.63) |
| Saint Kitts and Nevis | 4.08(0.2 to 13.17) | 2.85(0.14 to 9.53) | 11.42(0.56 to 36.87) | 5.18(0.26 to 17.26) | -2.92 (-3.18 to -2.67) |
| Saint Lucia | 5.72(0.27 to 18.72) | 7.17(0.36 to 23.69) | 7.31(0.35 to 23.52) | 3.51(0.18 to 11.38) | -3.13 (-3.56 to -2.71) |
| Saint Vincent and the Grenadines | 3.36(0.17 to 10.77) | 4.46(0.23 to 14.92) | 5.01(0.25 to 16) | 3.55(0.19 to 11.89) | -1.33 (-1.6 to -1.07) |
| Samoa | 3.06(0.3 to 12.38) | 5.82(0.44 to 19.77) | 4.52(0.38 to 17.28) | 4.88(0.33 to 16.13) | 0.02 (-0.15 to 0.2) |
| San Marino | 0.6(0.04 to 2.15) | 0.93(0.05 to 3.47) | 1.89(0.11 to 6.72) | 1.2(0.07 to 4.39) | -1.5 (-1.69 to -1.32) |
| Sao Tome and Principe | 3.05(0.13 to 11.6) | 5.45(0.2 to 20.19) | 5.31(0.23 to 20.61) | 5.87(0.23 to 21.88) | 0.38 (0.26 to 0.49) |
| Saudi Arabia | 132.39(16.87 to 539.28) | 282.28(31.99 to 1106.59) | 2.53(0.38 to 10.23) | 1.8(0.27 to 7.34) | -1.14 (-1.29 to -0.99) |
| Senegal | 151.12(5.67 to 586.01) | 316.61(11.42 to 1178.25) | 5.23(0.21 to 20.03) | 4.8(0.18 to 18.03) | -0.09 (-0.22 to 0.05) |
| Serbia | 4914.26(2038.71 to 8105.41) | 4255.78(1347.96 to 7959.62) | 50.94(20.76 to 84.04) | 28.08(8.86 to 52.6) | -2.42 (-2.64 to -2.19) |
| Seychelles | 8.55(2.06 to 16.84) | 6.87(0.64 to 15.65) | 15.17(3.56 to 30.02) | 6.85(0.65 to 15.61) | -2.75 (-3.07 to -2.43) |
| Sierra Leone | 104.72(3.75 to 412.65) | 186.92(6.07 to 693.89) | 5.99(0.22 to 23.98) | 5.86(0.21 to 21.94) | 0.24 (0.08 to 0.4) |
| Singapore | 213.39(43.35 to 419.65) | 159.32(23.68 to 355.95) | 10.87(2.07 to 21.7) | 2.14(0.32 to 4.82) | -6.04 (-6.23 to -5.85) |
| Slovakia | 1496.65(666.41 to 2396.74) | 975.61(320.62 to 1792.97) | 25.77(11.38 to 41.18) | 10.63(3.5 to 19.52) | -3.13 (-3.23 to -3.02) |
| Slovenia | 570.06(231.07 to 994.82) | 330.48(99.83 to 639.13) | 23.96(9.69 to 41.83) | 6.58(2.04 to 12.71) | -4.87 (-5.15 to -4.58) |
| Solomon Islands | 28.52(3.34 to 71.44) | 63.26(4.89 to 165.57) | 27(3.3 to 65.73) | 26.55(2.03 to 67.23) | -0.2 (-0.28 to -0.11) |
| Somalia | 496.55(27.31 to 1296.14) | 747.06(21.14 to 2194.19) | 22.07(1.35 to 57.49) | 13.91(0.4 to 40.84) | -1.6 (-1.68 to -1.51) |
| South Africa | 730.08(37.2 to 2725.78) | 959.81(74.88 to 4044.71) | 3.51(0.2 to 13.37) | 2.41(0.2 to 10.06) | -1.35 (-1.85 to -0.84) |
| South Sudan | 353.29(22.62 to 896.58) | 275.75(7.37 to 806.09) | 16.41(1.06 to 40.94) | 8.96(0.24 to 25.81) | -2.24 (-2.37 to -2.11) |
| Spain | 809.75(110.45 to 3232.43) | 636.92(92 to 2475.86) | 1.55(0.21 to 6.18) | 0.61(0.08 to 2.21) | -3.32 (-3.51 to -3.12) |
| Sri Lanka | 1408.47(358.18 to 2695.16) | 1565.45(199.08 to 3658.44) | 15.58(3.49 to 30.66) | 6.82(0.83 to 16.35) | -2.55 (-2.79 to -2.31) |
| Sudan | 277.92(32.19 to 1189.54) | 366.92(47.08 to 1522.17) | 3.15(0.43 to 13.53) | 2.16(0.31 to 9.08) | -1.42 (-1.47 to -1.36) |
| Suriname | 12.88(0.63 to 43.07) | 24(1.18 to 82.71) | 5.32(0.26 to 17.53) | 4.2(0.21 to 14.4) | -1.21 (-1.64 to -0.77) |
| Sweden | 371.19(24.2 to 1217.92) | 296.31(19.6 to 1018.65) | 2.31(0.15 to 7.42) | 1.19(0.08 to 3.96) | -2.63 (-2.77 to -2.49) |
| Switzerland | 228.15(15.66 to 780.95) | 155.25(10.9 to 550.86) | 2.05(0.14 to 6.98) | 0.74(0.05 to 2.59) | -3.62 (-3.83 to -3.4) |
| Syrian Arab Republic | 120.98(15.95 to 486.1) | 174.08(22.47 to 727.87) | 2.43(0.36 to 9.93) | 1.64(0.25 to 6.85) | -2.03 (-2.32 to -1.73) |
| Taiwan (Province of China) | 1828.46(253.72 to 4017.29) | 1331.32(119.35 to 3279.33) | 13.6(1.94 to 29.76) | 3.33(0.3 to 8.21) | -5.34 (-5.66 to -5.03) |
| Tajikistan | 365.06(66.63 to 810.2) | 398.47(28.05 to 1131.26) | 13.5(2.43 to 30.09) | 11.87(0.87 to 32.4) | -0.55 (-0.91 to -0.19) |
| Thailand | 4915.93(1356.17 to 9459.52) | 5513.94(694.93 to 12925.27) | 15.09(3.79 to 29.7) | 5.47(0.68 to 12.89) | -4.16 (-4.39 to -3.93) |
| Timor-Leste | 48.68(12.6 to 95.87) | 142.64(20.58 to 313.48) | 20.29(4.61 to 40.96) | 18.96(2.49 to 42.48) | -0.36 (-0.5 to -0.21) |
| Togo | 62.82(2.41 to 247.76) | 172.24(5.74 to 667.18) | 5.72(0.24 to 22.34) | 5.47(0.21 to 20.77) | -0.01 (-0.13 to 0.1) |
| Tokelau | 0.2(0.02 to 0.48) | 0.12(0.01 to 0.31) | 14.44(1.56 to 35.66) | 9.83(0.73 to 24.34) | -1.53 (-1.64 to -1.43) |
| Tonga | 3.92(0.47 to 9.27) | 5.2(0.42 to 12.4) | 8.37(0.96 to 19.73) | 6.9(0.56 to 16.37) | -0.77 (-0.96 to -0.58) |
| Trinidad and Tobago | 40.65(2.06 to 135.87) | 50.34(2.36 to 168.85) | 5.27(0.28 to 17.53) | 2.79(0.13 to 9.36) | -2.72 (-2.96 to -2.47) |
| Tunisia | 72.89(9.38 to 294.08) | 140.57(21.4 to 569.03) | 1.65(0.27 to 6.73) | 1.21(0.2 to 4.87) | -1.27 (-1.38 to -1.16) |
| Turkey | 275.81(60.43 to 1187.88) | 558.15(132.75 to 2378.48) | 0.85(0.2 to 3.67) | 0.67(0.16 to 2.83) | -0.16 (-0.55 to 0.22) |
| Turkmenistan | 243.7(44.3 to 538.76) | 368.9(29.58 to 1020.65) | 14.05(2.51 to 31.03) | 10.2(0.8 to 28.42) | -1.18 (-1.55 to -0.82) |
| Tuvalu | 1.12(0.13 to 2.82) | 1.24(0.1 to 3.03) | 18.67(2.16 to 46.86) | 13.34(1.02 to 32.69) | -1.37 (-1.45 to -1.28) |
| Uganda | 893.84(53.07 to 2256.85) | 1112.36(33.13 to 3281.62) | 15.74(0.95 to 39.01) | 9.76(0.29 to 28.22) | -2.22 (-2.51 to -1.92) |
| Ukraine | 3424.68(265.88 to 12009.48) | 2727.07(211.64 to 9698.07) | 5.07(0.41 to 17.8) | 3.66(0.28 to 12.75) | -2.05 (-2.38 to -1.72) |
| United Arab Emirates | 12.14(1.06 to 49.32) | 61.9(4.66 to 240.5) | 3.26(0.43 to 14.03) | 1.67(0.22 to 6.61) | -2.59 (-2.99 to -2.18) |
| United Kingdom | 2336.65(194.29 to 8449.91) | 1518.46(134.57 to 5402.18) | 2.49(0.21 to 8.99) | 1.06(0.09 to 3.7) | -3.32 (-3.55 to -3.09) |
| United Republic of Tanzania | 1698.83(135.96 to 3981.43) | 2971.74(149.99 to 7253.11) | 17.24(1.41 to 40.33) | 14.34(0.78 to 34.91) | -0.47 (-0.62 to -0.31) |
| United States of America | 4933.79(356.78 to 17031.51) | 7838.15(538.59 to 24009.36) | 1.5(0.11 to 5.17) | 1.35(0.09 to 4.08) | -0.55 (-0.73 to -0.37) |
| United States Virgin Islands | 2.1(0.1 to 7.05) | 4.2(0.2 to 14.33) | 2.99(0.14 to 9.83) | 2.42(0.12 to 8.12) | -0.53 (-0.72 to -0.34) |
| Uruguay | 270.56(16.38 to 713.71) | 213.58(10.94 to 572.53) | 7.09(0.44 to 18.46) | 3.57(0.19 to 9.45) | -2.82 (-3.13 to -2.51) |
| Uzbekistan | 1418.78(266.87 to 3163.94) | 1662.74(123.23 to 4551.27) | 13.4(2.5 to 29.67) | 12.46(1.02 to 32.57) | -0.86 (-1.6 to -0.1) |
| Vanuatu | 10.9(1.19 to 26.43) | 27.33(2.53 to 69.53) | 20.88(2.31 to 48.8) | 18.28(1.63 to 45.87) | -0.81 (-0.95 to -0.67) |
| Venezuela (Bolivarian Republic of) | 559.53(28.36 to 1421.09) | 1320.81(93.78 to 3332.51) | 6.16(0.3 to 15.87) | 4.68(0.33 to 11.77) | -1.35 (-1.54 to -1.17) |
| Viet Nam | 10802.28(2848.52 to 21066.09) | 16679.71(2459.87 to 35779.92) | 28.18(6.96 to 55.64) | 19.67(2.66 to 42.97) | -0.98 (-1.15 to -0.81) |
| Yemen | 128.35(14.72 to 522.41) | 261.91(35.68 to 1038.67) | 2.85(0.4 to 11.89) | 2.16(0.35 to 8.79) | -1.2 (-1.3 to -1.11) |
| Zambia | 417.89(24.72 to 1067.56) | 776.24(24.41 to 2303.88) | 17.24(1.07 to 43.53) | 14.55(0.46 to 41.82) | -0.82 (-0.96 to -0.68) |
| Zimbabwe | 156.57(5.84 to 559.39) | 298.75(10.3 to 1087.6) | 4.48(0.19 to 16.59) | 4.93(0.19 to 18.92) | 0.89 (0.63 to 1.15) |

ASMR, age-standard morality rate; EAPC, estimated annual percentage change.
