# Supplementary material for: Global, regional, and national burden of stroke attributable to diet high in sodium from 1990 to 2019: a systematic analysis from the global burden of disease study 2019
Source: Front Neurol. 2024 Aug 14;15:1437633. doi: 10.3389/fneur.2024.1437633 (PMC11349671; doi:10.3389/fneur.2024.1437633)
Supplement: Supplementary Table S2 — DALYs number and age-standardized DALYs rate of Stroke attributable to Diet high in sodium for both sexes combined in 1990 and 2019, and EAPC of ASMR from 1990 to 2019 in 204 countries and territories. [file Table_2.docx]

Table 2S. DALYs number and age-standardized DALYs rate of Stroke attributable to Diet high in sodium for both sexes combined in 1990 and 2019, and EAPC of ASMR from 1990 to 2019 in 204 countries and territories

| Location | DALYs number in 1990 | DALYs number in 2019 | ASDR in 1990 | ASDR in 2019 | EAPC 1990-2019 |
| --- | --- | --- | --- | --- | --- |
| Afghanistan | 6354.14(699.17 to 27339.93) | 9932.83(1083.7 to 41823.27) | 87(10.26 to 372) | 65.63(7.95 to 274.61) | -1.15 (-1.41 to -0.9) |
| Albania | 13573.91(6171.89 to 21865.1) | 16377.59(5001.97 to 31148.14) | 702.06(316.91 to 1130.25) | 383.75(119.29 to 726.53) | -2.15 (-2.35 to -1.95) |
| Algeria | 7018.39(789.01 to 28631.15) | 10258.49(1346.71 to 39675.15) | 56.6(7.98 to 234.21) | 30.43(4.5 to 118.17) | -2.33 (-2.41 to -2.24) |
| American Samoa | 19.61(1.45 to 82.87) | 38.18(2.26 to 144.83) | 93.11(6.23 to 365.83) | 87.17(4.85 to 310.85) | -0.52 (-0.66 to -0.38) |
| Andorra | 12.69(0.74 to 45.22) | 20.98(1.34 to 73.76) | 23.39(1.48 to 84.44) | 15.04(0.94 to 52.76) | -1.58 (-1.77 to -1.39) |
| Angola | 5641.28(261.61 to 23113.93) | 12527.91(507.6 to 50623.85) | 131.62(6.6 to 555.18) | 108.83(4.7 to 438.3) | -0.88 (-1.04 to -0.71) |
| Antigua and Barbuda | 50.86(2.4 to 167.96) | 54.96(2.76 to 185.88) | 95.02(4.45 to 315.63) | 55.02(2.81 to 185.66) | -2.24 (-2.49 to -1.98) |
| Argentina | 46113.28(2454.48 to 126542.08) | 34218.08(1653.74 to 95553.75) | 143.72(7.74 to 394.61) | 64.76(3.12 to 181.32) | -3.16 (-3.42 to -2.89) |
| Armenia | 6035.7(1111.13 to 13337.89) | 3371.37(261.2 to 9095.74) | 234.13(43.52 to 515.74) | 83.63(6.47 to 223.6) | -4.33 (-4.63 to -4.02) |
| Australia | 5041.83(466.08 to 19267.68) | 4476.48(410.28 to 17300.57) | 26.57(2.43 to 101.19) | 11.69(0.96 to 43.22) | -3.07 (-3.26 to -2.88) |
| Austria | 10032.15(509.13 to 28657.62) | 5372.89(291.96 to 14597.99) | 83.23(4.22 to 236.4) | 29.4(1.6 to 78.77) | -3.92 (-4.17 to -3.68) |
| Azerbaijan | 13859.83(2409.32 to 31285.37) | 16036.78(1129.71 to 44869.49) | 272.35(47.86 to 615.12) | 193.41(14.61 to 529.98) | -0.85 (-1.17 to -0.54) |
| Bahamas | 125.27(6.05 to 436.36) | 223.09(10.41 to 772.18) | 80.31(3.82 to 277.35) | 56(2.67 to 193.25) | -1.41 (-1.56 to -1.25) |
| Bahrain | 77.52(7.83 to 302.01) | 191.97(19.54 to 740.03) | 36.62(4.81 to 147.38) | 17.09(2.33 to 68.44) | -2.81 (-2.99 to -2.62) |
| Bangladesh | 77993.49(3601.63 to 273079.27) | 219667.78(10272.14 to 622186.71) | 160.98(7.54 to 565.8) | 168.04(7.95 to 480.14) | 1.11 (0.61 to 1.62) |
| Barbados | 155.8(11.61 to 617.11) | 174.24(11.46 to 681.04) | 54.68(3.95 to 215.15) | 36.34(2.39 to 140.55) | -1.73 (-2 to -1.46) |
| Belarus | 12982.28(829.8 to 43632.08) | 11666.01(718.92 to 41576.7) | 100.3(6.52 to 337.67) | 76.15(4.6 to 270.23) | -1.67 (-2.2 to -1.14) |
| Belgium | 12154.55(616.62 to 33094.19) | 7483.71(340.66 to 21396.65) | 78.58(3.9 to 214.82) | 32.23(1.44 to 93.59) | -3.27 (-3.38 to -3.15) |
| Belize | 59.13(2.9 to 201.99) | 138.59(6.76 to 470.39) | 64.1(3.11 to 217.92) | 48.98(2.42 to 164.23) | -1.42 (-1.83 to -1.02) |
| Benin | 3795.63(118.21 to 13137.29) | 7723.48(221.84 to 26394.86) | 187.63(5.94 to 646.44) | 154.6(4.65 to 520.39) | -0.68 (-0.75 to -0.61) |
| Bermuda | 37.2(1.77 to 122.64) | 33.01(1.59 to 108.74) | 60.81(2.9 to 201.83) | 25.74(1.24 to 84.78) | -3.11 (-3.3 to -2.92) |
| Bhutan | 228.22(9.63 to 823.98) | 476.93(22.39 to 1377.66) | 91.55(4.01 to 326.43) | 85.24(3.98 to 243.61) | 0.2 (-0.01 to 0.42) |
| Bolivia (Plurinational State of) | 5376.28(202.6 to 15509.94) | 7754.41(353.5 to 23081.36) | 159.61(6.14 to 457.85) | 86.71(4.02 to 256.18) | -2.32 (-2.53 to -2.11) |
| Bosnia and Herzegovina | 23381.67(9707.94 to 37742.68) | 21980.06(6701.2 to 41567.97) | 604.52(252.18 to 976.62) | 373.54(112.75 to 702.38) | -1.95 (-2.13 to -1.77) |
| Botswana | 1073.29(37.25 to 3993.47) | 1645.75(63.4 to 6614.85) | 171.65(6.67 to 644.05) | 106.13(4.93 to 439.76) | -2.12 (-2.48 to -1.76) |
| Brazil | 206121.91(11281.25 to 545878.2) | 201535.25(12471.27 to 533170.17) | 220.97(12.21 to 582.69) | 84.39(5.25 to 224.59) | -3.56 (-3.68 to -3.45) |
| Brunei Darussalam | 484.72(104.88 to 952.63) | 536.91(87.83 to 1112.05) | 485.61(102.67 to 963.82) | 178.77(28.15 to 382.66) | -3.8 (-4.03 to -3.56) |
| Bulgaria | 128654.57(57423.52 to 204391.69) | 82729.82(25891.67 to 154273.36) | 1048.01(468.36 to 1656.49) | 570(180.89 to 1066.48) | -2.7 (-2.91 to -2.49) |
| Burkina Faso | 4725.38(165.42 to 18524.92) | 10697.25(351.55 to 40764.56) | 103.05(3.81 to 405.94) | 109.98(3.78 to 420.42) | 0.45 (0.39 to 0.5) |
| Burundi | 14317.06(721.67 to 38904.33) | 11072.39(312.04 to 34250.62) | 590.95(30.17 to 1574.39) | 247.81(7.15 to 740.84) | -3.49 (-3.69 to -3.3) |
| Cabo Verde | 174.17(6.77 to 664.29) | 397.33(13.46 to 1452.49) | 79.5(3.01 to 302.05) | 90.17(3.11 to 330.84) | -0.14 (-0.35 to 0.07) |
| Cambodia | 31329.13(8656.35 to 58928.71) | 42037.84(5389.45 to 92292.14) | 667.63(180.64 to 1256.35) | 351.44(43.14 to 776.14) | -2.59 (-2.74 to -2.44) |
| Cameroon | 5960.14(213.72 to 23826.5) | 16630.37(506.9 to 64849.7) | 125.25(4.75 to 496.72) | 129.46(4.21 to 495.42) | 0.27 (-0.04 to 0.57) |
| Canada | 15457.79(857.15 to 45900.79) | 16917.14(972.17 to 49608.03) | 47.89(2.66 to 141.78) | 26.16(1.53 to 75.14) | -2.47 (-2.64 to -2.29) |
| Central African Republic | 2030(98.85 to 8848.93) | 3857.87(144.79 to 16388.95) | 159.7(8.2 to 707) | 163.39(6.47 to 698.04) | 0.05 (-0.04 to 0.13) |
| Chad | 3733.37(136.36 to 14464.75) | 8121.26(258.29 to 31023.32) | 129.94(4.84 to 494.05) | 136.28(4.51 to 520.82) | 0.3 (0.14 to 0.46) |
| Chile | 14799.38(882.06 to 37510.09) | 15140.4(728.52 to 39398.8) | 147.63(8.88 to 374.15) | 63.51(3.08 to 165.32) | -2.88 (-2.98 to -2.77) |
| China | 8115351.99(4038246.68 to 12929119.59) | 9990177.02(4491757.74 to 16396841.48) | 916.34(432.4 to 1488.53) | 486.13(214.74 to 807.53) | -2.22 (-2.34 to -2.09) |
| Colombia | 35238.81(7146.7 to 73240.6) | 42055.14(9099.93 to 89200.23) | 193.27(40.28 to 400.59) | 79.45(17.23 to 169.29) | -3.54 (-3.71 to -3.37) |
| Comoros | 949.37(47.01 to 2482.28) | 990.85(30.46 to 2813.54) | 422.6(21.96 to 1088.23) | 208.48(6.42 to 579.42) | -2.81 (-3.02 to -2.59) |
| Congo | 1766.9(87.31 to 7509.52) | 3156.37(125.05 to 12758.43) | 157.23(8.02 to 671.35) | 117.18(4.96 to 458.8) | -1.3 (-1.46 to -1.13) |
| Cook Islands | 25.53(3.02 to 63.01) | 32.93(2.57 to 80.68) | 212.26(25.75 to 512.79) | 129.4(10.33 to 322.33) | -2.03 (-2.22 to -1.84) |
| Costa Rica | 1459.18(73.35 to 3664.87) | 2473.59(191.05 to 6162.8) | 81.34(4.03 to 205.17) | 47.85(3.73 to 119.14) | -2.21 (-2.54 to -1.88) |
| Croatia | 42744.08(18687.26 to 67963.48) | 20800.06(6668.05 to 37856.97) | 680.69(299.77 to 1079.53) | 231.7(74.04 to 420.79) | -4.15 (-4.33 to -3.97) |
| Cuba | 6505.76(326.35 to 21816.66) | 7931.64(402.03 to 27062.72) | 63.56(3.18 to 213.33) | 42.06(2.12 to 142.65) | -1.71 (-1.88 to -1.54) |
| Cyprus | 502.5(28.45 to 1671.78) | 505.43(29.38 to 1630.02) | 68.35(4.04 to 228.41) | 27.7(1.63 to 89.55) | -3.64 (-3.8 to -3.48) |
| Czechia | 92552.12(40232.02 to 151338.61) | 31353.61(10218.26 to 57911.2) | 669.71(289.97 to 1089.66) | 148.12(47.69 to 274.25) | -5.46 (-5.67 to -5.25) |
| C么te d'Ivoire | 7351.14(219.09 to 26750.68) | 15212.01(423.99 to 55499.98) | 161.79(5.23 to 582.77) | 130.01(3.96 to 472.21) | -0.86 (-1.05 to -0.67) |
| Democratic People's Republic of Korea | 168771.77(72895.15 to 277201.41) | 216163.59(63343.32 to 408249.04) | 972.85(410.34 to 1637.6) | 667.85(196.55 to 1262.67) | -1.44 (-1.64 to -1.23) |
| Democratic Republic of the Congo | 13124.72(951.61 to 61499.83) | 22734.82(1707.94 to 112037.88) | 78.99(6.38 to 370.04) | 60.47(4.95 to 303.89) | -0.93 (-1 to -0.87) |
| Denmark | 4768.06(252.9 to 15623.58) | 3078.47(161.08 to 9910.57) | 61.54(3.14 to 197.29) | 28.4(1.42 to 89.9) | -3.08 (-3.25 to -2.91) |
| Djibouti | 565.07(28.91 to 1494.92) | 1195.57(35.63 to 3600.34) | 381.39(20.98 to 977.14) | 209.3(6.24 to 607.92) | -2.33 (-2.41 to -2.24) |
| Dominica | 53.16(2.58 to 177.38) | 50.87(2.5 to 175.24) | 74.38(3.64 to 251.83) | 56.7(2.78 to 196.41) | -0.81 (-0.99 to -0.64) |
| Dominican Republic | 2808.4(136.41 to 9527.99) | 7985.35(355 to 28123.14) | 74.53(3.59 to 250.02) | 84.79(3.83 to 294.65) | 1.02 (0.8 to 1.24) |
| Ecuador | 5975.56(236.57 to 16080.25) | 8751.69(415.4 to 25101.8) | 105.52(4.17 to 283.06) | 57.34(2.72 to 164.47) | -2.14 (-2.42 to -1.85) |
| Egypt | 14976.13(1573.97 to 58512.82) | 28056.21(2990.35 to 112004.23) | 45.12(5.31 to 181.36) | 39.62(4.69 to 160.31) | -0.14 (-0.25 to -0.02) |
| El Salvador | 4080.04(240.56 to 10252.8) | 3457.62(251.3 to 9291.94) | 130.68(7.51 to 327.81) | 58.22(4.18 to 156.44) | -3.11 (-3.62 to -2.6) |
| Equatorial Guinea | 298.04(14.31 to 1314.72) | 400.43(15.18 to 1596.74) | 143.48(7.14 to 636.82) | 82.54(3.41 to 327.59) | -2.33 (-2.59 to -2.07) |
| Eritrea | 5367.75(267.07 to 14210.4) | 7038.41(223.04 to 21665.24) | 489.72(27.48 to 1283.7) | 269.41(8.32 to 797.1) | -2.27 (-2.36 to -2.18) |
| Estonia | 1551.01(173.77 to 5768.5) | 460.57(52.96 to 1741.34) | 75.8(8.68 to 279.87) | 19(2.05 to 70.67) | -6.12 (-6.66 to -5.57) |
| Eswatini | 505.47(18.08 to 1888.81) | 712.01(27.39 to 2878.39) | 156.36(6.33 to 612.23) | 112.01(5.02 to 468.01) | -0.81 (-1.17 to -0.45) |
| Ethiopia | 98090.22(7435.42 to 253318.19) | 64784.87(2868.69 to 190272.87) | 460.62(37.16 to 1184.35) | 163.66(7.12 to 474.66) | -4.01 (-4.19 to -3.82) |
| Fiji | 876.13(98.45 to 2325.03) | 1495.97(113.66 to 3881.9) | 278.55(32.5 to 679.24) | 209.47(16.91 to 525.54) | -1.63 (-1.92 to -1.35) |
| Finland | 5199.17(272.33 to 15938.69) | 3402.39(209.74 to 10689.77) | 76.37(3.94 to 231.94) | 31.29(1.82 to 95.32) | -3.2 (-3.37 to -3.03) |
| France | 29131.49(1840.77 to 104031.73) | 23010.13(1387.52 to 79531.6) | 34.96(2.18 to 126.2) | 17.45(1 to 58.71) | -2.35 (-2.48 to -2.22) |
| Gabon | 630.64(33.23 to 2771.89) | 944.69(39.29 to 3765.67) | 112.68(5.99 to 486.46) | 90.89(3.94 to 358.41) | -0.87 (-1.06 to -0.67) |
| Gambia | 449.58(15.23 to 1760.13) | 1305.72(43.66 to 4932.75) | 119.37(4.27 to 469.13) | 132.03(4.52 to 500.43) | 0.41 (0.25 to 0.57) |
| Georgia | 29004.06(5318.59 to 62937.64) | 13132(1002.72 to 35015.85) | 480.23(90.73 to 1033.54) | 225.05(18.1 to 593.89) | -2.75 (-3.04 to -2.45) |
| Germany | 80997.49(4677.99 to 255892.82) | 47860.52(2931.92 to 147782.96) | 65.88(3.71 to 202.29) | 27.45(1.63 to 81.37) | -3.34 (-3.68 to -3) |
| Ghana | 12470.28(367.69 to 45821.49) | 37097.11(924.65 to 119677.69) | 186.51(5.9 to 682.04) | 218.55(5.75 to 688.12) | 1.31 (0.98 to 1.63) |
| Greece | 12420.52(736.02 to 41825.21) | 10042.85(629.08 to 32698.78) | 83.52(4.94 to 277.4) | 43.46(2.51 to 136.74) | -2.73 (-2.93 to -2.54) |
| Greenland | 56.73(2.83 to 173.49) | 60.43(3.13 to 177.3) | 151.67(8.16 to 484.15) | 86.29(4.77 to 259.28) | -2.24 (-2.39 to -2.09) |
| Grenada | 100.81(4.99 to 334.51) | 81.27(4.07 to 284.64) | 139.66(6.88 to 473.24) | 73.53(3.74 to 251.72) | -2.31 (-2.51 to -2.11) |
| Guam | 118.86(12.77 to 296.33) | 221.61(17.52 to 548.41) | 178.73(20.75 to 418.81) | 116.36(9.28 to 288.72) | -1.91 (-2.18 to -1.64) |
| Guatemala | 4108.82(228.58 to 10779.07) | 8556.57(505.84 to 22505.6) | 104.31(5.68 to 272.8) | 73.55(4.05 to 192.66) | -1.87 (-2.2 to -1.55) |
| Guinea | 4251.49(156.3 to 16635.22) | 7816.51(254.24 to 29374.08) | 125.27(4.73 to 488.25) | 135.93(4.59 to 510.33) | 0.72 (0.58 to 0.86) |
| Guinea-Bissau | 878.39(29.19 to 3506.74) | 1450.55(45.73 to 5346.16) | 199.14(6.91 to 781.15) | 180.5(5.96 to 666.67) | -0.08 (-0.19 to 0.03) |
| Guyana | 1072.38(50.33 to 3711.95) | 852.03(39.65 to 3030.15) | 276.79(12.99 to 955.46) | 134.09(6.42 to 466.81) | -2.5 (-2.75 to -2.24) |
| Haiti | 6797.12(306.18 to 25352.72) | 10314.23(432.14 to 39554.08) | 206.25(9.11 to 751.22) | 143.25(6.13 to 548.88) | -1.13 (-1.27 to -0.98) |
| Honduras | 3203.36(175.88 to 8288.34) | 10286.32(664.41 to 27087.04) | 142.99(7.63 to 366.31) | 166.8(11.18 to 437.93) | 0.66 (0.51 to 0.8) |
| Hungary | 128059.31(67219.33 to 189704.7) | 48263.25(18640.78 to 82058.51) | 882.59(466.89 to 1303) | 256.39(101.08 to 437.02) | -4.9 (-5.13 to -4.66) |
| Iceland | 132.54(6.91 to 408.1) | 111.02(5.52 to 330.1) | 46.52(2.39 to 141.14) | 20.23(0.98 to 59.52) | -3.24 (-3.39 to -3.08) |
| India | 718176.62(54444.03 to 1990121.77) | 1268329.49(110759.02 to 3360900.85) | 146.38(10.65 to 417.11) | 104.55(8.92 to 283.89) | -1.12 (-1.23 to -1.02) |
| Indonesia | 716990.75(226351.71 to 1266278.21) | 1142815.35(198759.99 to 2368483.68) | 690.41(212.86 to 1232) | 519.67(91.81 to 1072.34) | -0.94 (-0.99 to -0.9) |
| Iran (Islamic Republic of) | 12111.74(1650.42 to 46296.07) | 17916.8(2877.71 to 69015.61) | 44.71(7.21 to 170.28) | 23.94(4.1 to 93.38) | -2.36 (-2.52 to -2.2) |
| Iraq | 6266.47(666.25 to 25514.44) | 15389.28(1677.59 to 59694.87) | 75.58(8.61 to 312.48) | 61.89(7.58 to 244.57) | -0.87 (-0.96 to -0.78) |
| Ireland | 1808.3(124.14 to 6441.19) | 1159.18(82.79 to 4145.81) | 44.72(3.08 to 160.07) | 15.97(1.11 to 56.61) | -3.99 (-4.23 to -3.75) |
| Israel | 2352.71(126.55 to 7656.24) | 2490.28(138.47 to 7667.86) | 49.32(2.63 to 159.65) | 22.14(1.22 to 67.82) | -3.31 (-3.54 to -3.07) |
| Italy | 79030.64(7006.38 to 210270.47) | 43151.19(3553.8 to 121549.68) | 91.75(8.66 to 240.21) | 32.58(2.95 to 87.19) | -3.72 (-4.03 to -3.42) |
| Jamaica | 1652.92(84.23 to 5718.56) | 2116.87(104.64 to 7304.36) | 92.62(4.71 to 322.08) | 70.7(3.46 to 242.89) | -0.94 (-1.33 to -0.55) |
| Japan | 438870.55(151414.93 to 760838.92) | 223712.48(28051.69 to 508340.54) | 261.34(88.36 to 454.43) | 71.39(8.93 to 161.39) | -5.08 (-5.31 to -4.84) |
| Jordan | 695.99(89.69 to 2770.6) | 1730.37(215.84 to 6757.8) | 51.94(7.57 to 208.24) | 26.28(3.8 to 103.29) | -2.95 (-3.25 to -2.66) |
| Kazakhstan | 47391.87(8630.84 to 101593.93) | 36555.28(2897.43 to 98957.49) | 375.52(66.51 to 807.46) | 218.49(17.03 to 587.33) | -2.57 (-2.92 to -2.23) |
| Kenya | 14616.9(1336.5 to 40888.2) | 27722.5(2664.63 to 81912.23) | 178.96(16.62 to 496.77) | 131.13(12.24 to 377.11) | -1.01 (-1.07 to -0.95) |
| Kiribati | 205.77(21.2 to 560.31) | 322.13(24.27 to 869.8) | 560.5(60.06 to 1446.46) | 465.65(37.27 to 1194.31) | -0.98 (-1.14 to -0.82) |
| Kuwait | 296.59(16.24 to 940.44) | 1128.85(58.08 to 3526.8) | 37.55(2.37 to 127.78) | 36.07(2.01 to 120.82) | 0.55 (-0.09 to 1.19) |
| Kyrgyzstan | 11751.64(2027.68 to 25992.82) | 7813.09(586.92 to 21183.87) | 386.81(69.5 to 853.33) | 159.89(12.17 to 434.06) | -3.81 (-4.25 to -3.37) |
| Lao People's Democratic Republic | 17303.92(4837.73 to 32543.96) | 20224.68(2847.01 to 43701.96) | 787.72(213.95 to 1491.77) | 449.28(63.72 to 970.15) | -2.28 (-2.47 to -2.1) |
| Latvia | 5703.07(382.33 to 17434.44) | 3191.26(209.36 to 10149.87) | 159.72(10.85 to 487.85) | 84.57(5.31 to 259.78) | -3 (-3.39 to -2.6) |
| Lebanon | 439.79(57.32 to 1824.06) | 699.73(100.46 to 2900.68) | 19.56(2.85 to 79.79) | 13.2(1.92 to 54.86) | -1.17 (-1.36 to -0.98) |
| Lesotho | 1430.31(53.66 to 5567.69) | 2031.05(79.3 to 8076.85) | 139.49(5.64 to 542.8) | 147.84(6.5 to 591.8) | 0.91 (0.63 to 1.2) |
| Liberia | 1469.14(52.13 to 5531.97) | 2342.04(73.26 to 8564.94) | 130.52(4.76 to 492.4) | 105.85(3.57 to 382.89) | -0.67 (-0.82 to -0.52) |
| Libya | 674.93(78.39 to 2720.67) | 1693(192.68 to 6548.88) | 33.63(4.21 to 136.25) | 30.14(3.71 to 121.9) | -0.41 (-0.57 to -0.26) |
| Lithuania | 4247.17(210.72 to 13702.89) | 3572.18(175.76 to 11793.36) | 94.32(4.69 to 303.72) | 64.32(3.05 to 210.04) | -1.51 (-1.81 to -1.21) |
| Luxembourg | 403.89(23.27 to 1326.52) | 229.29(13.48 to 715.58) | 75.42(4.3 to 245.09) | 23.24(1.33 to 71.02) | -4.24 (-4.36 to -4.13) |
| Madagascar | 30289.09(1680.48 to 77588.08) | 41500.99(1189.81 to 129726.82) | 553.8(33.15 to 1394.89) | 370.98(10.41 to 1091.73) | -1.67 (-1.81 to -1.52) |
| Malawi | 14727.84(858.72 to 37391.39) | 15017.45(473.02 to 43519.63) | 375.23(22.91 to 944.46) | 214.5(6.66 to 605.49) | -2.29 (-2.49 to -2.08) |
| Malaysia | 56899.41(16022.95 to 103264.99) | 64833.58(8987.47 to 138918.61) | 607.46(171.79 to 1094.19) | 238.25(31.84 to 513.41) | -3.2 (-3.46 to -2.94) |
| Maldives | 541.89(169.15 to 964.44) | 456.92(65.01 to 985.37) | 554.4(164.57 to 1003.06) | 149.3(21.75 to 315.14) | -5.32 (-5.68 to -4.96) |
| Mali | 5849.14(215.28 to 22900.79) | 9982.97(338.88 to 39067.26) | 135.47(5.18 to 528.29) | 112.68(4 to 432.47) | -0.61 (-0.74 to -0.48) |
| Malta | 329.19(18.4 to 985.28) | 276.51(17.52 to 784.31) | 77.73(4.38 to 232.93) | 33.15(2.24 to 91.02) | -2.97 (-3.14 to -2.8) |
| Marshall Islands | 64.24(7.3 to 160.35) | 114.59(8.93 to 312.51) | 414.31(45.03 to 1022.16) | 345.92(29.14 to 888.26) | -0.63 (-0.74 to -0.51) |
| Mauritania | 1546.97(57.17 to 6062.88) | 1859.2(61.9 to 7043.87) | 149.62(5.68 to 577.96) | 87.6(3.04 to 330.1) | -1.6 (-1.77 to -1.42) |
| Mauritius | 4595.38(1312.62 to 8377.32) | 3071.48(429.29 to 6621.29) | 602.58(167.88 to 1107.34) | 173.27(22.9 to 376.39) | -5.45 (-5.99 to -4.9) |
| Mexico | 27172.03(1328.86 to 80236.52) | 47723.27(2463.34 to 140622.13) | 61.82(3.05 to 184.42) | 40.27(2.12 to 119.13) | -1.66 (-1.8 to -1.52) |
| Micronesia (Federated States of) | 188.33(22.37 to 510.62) | 237.11(19.39 to 643.19) | 414.87(50.79 to 1067.89) | 344.52(30.33 to 890.34) | -0.89 (-0.98 to -0.8) |
| Monaco | 34.62(1.91 to 120.45) | 23.19(1.3 to 80) | 50.61(2.57 to 170.83) | 26.32(1.36 to 85.78) | -2.26 (-2.65 to -1.88) |
| Mongolia | 5462.26(960.08 to 12479.37) | 9814.94(704.26 to 27219.59) | 503.09(89.27 to 1158.58) | 363.76(27.94 to 1012.08) | -1.34 (-1.78 to -0.9) |
| Montenegro | 4883.18(2243.6 to 7933.98) | 5670.45(1793.47 to 10532.82) | 804.6(367.37 to 1317.58) | 584.44(187.36 to 1080.48) | -1.23 (-1.41 to -1.04) |
| Morocco | 7929.91(863.86 to 31888.15) | 13745.59(1648.81 to 54923.53) | 54.94(6.58 to 225.94) | 43.75(5.69 to 173.26) | -0.91 (-0.99 to -0.84) |
| Mozambique | 28822.28(1787.58 to 71894.67) | 38808.65(1141.83 to 116469.06) | 469.41(30.9 to 1155.33) | 359.88(10.41 to 1041.15) | -0.66 (-0.79 to -0.53) |
| Myanmar | 263965.31(76270.12 to 499619.22) | 239166.18(31118.43 to 510984.67) | 1071.63(305.06 to 2022.18) | 507.98(66.67 to 1076.17) | -2.96 (-3.13 to -2.78) |
| Namibia | 1213.41(47.19 to 4593.33) | 1381.23(62.27 to 5659.53) | 162.88(6.7 to 618.86) | 93.47(4.64 to 383.67) | -2.26 (-2.54 to -1.98) |
| Nauru | 14.56(1.61 to 42.44) | 15.81(1.09 to 46.86) | 465.86(54.84 to 1155.49) | 407.95(30.56 to 1056.33) | -0.71 (-1.22 to -0.2) |
| Nepal | 9541.53(412.37 to 34328.58) | 22286.91(1032.78 to 64737.88) | 98.12(4.4 to 348.02) | 99.52(4.69 to 287.31) | 0.69 (0.33 to 1.05) |
| Netherlands | 7007.08(491.19 to 25889.58) | 7175.94(430.59 to 24705.27) | 35.83(2.44 to 129.54) | 22.46(1.29 to 74.26) | -2.27 (-2.6 to -1.95) |
| New Zealand | 1852.32(101.16 to 6114.11) | 1571.54(92.65 to 5205.04) | 47.75(2.63 to 157.81) | 21.15(1.19 to 69.7) | -3.12 (-3.29 to -2.95) |
| Nicaragua | 2006.53(105.37 to 5060.44) | 3455.72(228.5 to 8636.31) | 127.05(6.49 to 319.9) | 79.1(5.07 to 201.87) | -2.08 (-2.27 to -1.9) |
| Niger | 4349.49(147.78 to 17066.27) | 10232.47(317.19 to 39386.22) | 142.45(5.12 to 557.64) | 124.66(4.14 to 471.65) | -0.42 (-0.5 to -0.34) |
| Nigeria | 53455.77(2313.09 to 213626.54) | 74016.92(3249.1 to 290546.21) | 119.74(5.39 to 483.26) | 83.4(3.76 to 320.34) | -1.42 (-1.55 to -1.28) |
| Niue | 6.61(0.81 to 15.77) | 5.05(0.38 to 12.59) | 301.11(36.85 to 725.09) | 231.67(18.3 to 581.09) | -1.32 (-1.49 to -1.15) |
| North Macedonia | 19412.51(8498.88 to 31582.93) | 21404.25(6610.9 to 39569.52) | 1097.91(477.31 to 1801.43) | 710.24(222.92 to 1311.81) | -1.84 (-1.99 to -1.7) |
| Northern Mariana Islands | 41.49(4.6 to 117.19) | 104.04(8.57 to 252.63) | 295.74(34.26 to 722.08) | 200.09(17.75 to 474.96) | -1.66 (-1.85 to -1.47) |
| Norway | 3419.69(261.17 to 11388.4) | 1954.12(151.55 to 6391.19) | 51.69(3.84 to 166.75) | 21(1.56 to 68.5) | -3.32 (-3.41 to -3.23) |
| Oman | 430.53(42.76 to 1822.89) | 658.24(69.02 to 2532.04) | 56.55(6.86 to 241.4) | 33.43(4.62 to 136.8) | -1.38 (-1.64 to -1.13) |
| Pakistan | 68472.65(3032.71 to 233253.19) | 193387.26(10157.59 to 534067.16) | 118.8(5.31 to 407.4) | 166.42(8.76 to 458.09) | 1.47 (1.22 to 1.72) |
| Palau | 28.13(3.4 to 70.98) | 53.52(4.8 to 133.53) | 290.14(34.18 to 714.77) | 246.84(23.48 to 602.45) | -0.73 (-0.83 to -0.63) |
| Palestine | 490.26(61.13 to 1980.57) | 854.78(111.42 to 3417.06) | 56.68(7.68 to 228.64) | 37.06(5.5 to 149.68) | -1.52 (-1.61 to -1.44) |
| Panama | 1815.08(109 to 4476.14) | 2870.76(223.37 to 7138.49) | 119.94(7.13 to 295.25) | 68.99(5.39 to 171.66) | -2.06 (-2.16 to -1.96) |
| Papua New Guinea | 4458.36(478.19 to 12683.69) | 11608.06(819.2 to 33534.18) | 265.72(30.15 to 697.45) | 262.72(20.51 to 700.46) | -0.12 (-0.26 to 0.02) |
| Paraguay | 3446.87(135.17 to 9408.98) | 5044.67(236.69 to 14809.1) | 152.88(5.9 to 417.01) | 89.68(4.19 to 259.82) | -2.14 (-2.3 to -1.98) |
| Peru | 11652.49(491.86 to 33028.26) | 12423.76(607.32 to 36346.22) | 93.25(3.9 to 259.47) | 37.91(1.84 to 111.23) | -3.23 (-3.57 to -2.89) |
| Philippines | 79317.65(23436.9 to 145610.23) | 252458.27(39900.36 to 551365.55) | 265.57(75.6 to 488.81) | 306.63(47.92 to 659.03) | 1.05 (0.45 to 1.66) |
| Poland | 102261.18(18061.23 to 224180.39) | 92918.52(16230.23 to 193974.11) | 239.05(42.58 to 521.9) | 137.25(24.5 to 286.02) | -2.15 (-2.24 to -2.06) |
| Portugal | 13892.32(1026.23 to 49270.12) | 8946.78(677.88 to 27353.98) | 103.37(7.66 to 363.43) | 42.32(3.13 to 123.25) | -3.39 (-3.56 to -3.22) |
| Puerto Rico | 1335.84(64.14 to 4350.29) | 1348.24(65.47 to 4448.91) | 37.36(1.79 to 122.31) | 19.73(0.95 to 65.66) | -2.76 (-2.99 to -2.53) |
| Qatar | 59.7(5.09 to 230.37) | 249.96(20.56 to 948.53) | 31.44(3.83 to 127.13) | 16.97(2.19 to 65.92) | -2.21 (-2.41 to -2.01) |
| Republic of Korea | 173221.48(35973.81 to 339346.04) | 100177.43(21121.11 to 201336.24) | 603.62(121.66 to 1188.22) | 114.06(23.56 to 229.88) | -6.45 (-6.73 to -6.17) |
| Republic of Moldova | 5546.9(338.94 to 19241.75) | 4311.28(255.09 to 15230.68) | 124.82(7.92 to 428.38) | 75.43(4.46 to 261.79) | -2.06 (-2.46 to -1.66) |
| Romania | 230193.06(101137.01 to 364717.4) | 154407.71(48385.18 to 271768.55) | 839.88(364.79 to 1343.59) | 412.75(130.74 to 733.3) | -3.32 (-3.65 to -2.98) |
| Russian Federation | 477552.16(48198.46 to 1212383.87) | 441616.93(45229.03 to 1112788.04) | 262.19(26.13 to 679.17) | 193.36(20.36 to 484.9) | -1.85 (-2.5 to -1.2) |
| Rwanda | 19252.65(1088.35 to 49630.92) | 12425.8(370.8 to 36617.66) | 636.4(36.68 to 1603.93) | 215.96(6.33 to 628.13) | -4.7 (-5.09 to -4.3) |
| Saint Kitts and Nevis | 78.6(3.88 to 263.31) | 65.58(2.91 to 226.85) | 216.29(10.63 to 719.88) | 100.66(4.75 to 340.2) | -3.12 (-3.42 to -2.82) |
| Saint Lucia | 113.89(5.47 to 385.11) | 143.92(6.92 to 482.38) | 133.68(6.43 to 447.19) | 67.29(3.26 to 227.3) | -2.78 (-3.18 to -2.39) |
| Saint Vincent and the Grenadines | 65.26(3.36 to 218.65) | 93.11(4.6 to 319.8) | 92.07(4.72 to 309.76) | 69.84(3.51 to 239.49) | -1.17 (-1.4 to -0.94) |
| Samoa | 63.29(8.27 to 279.61) | 110.55(12.3 to 420.56) | 79.96(9.27 to 342.74) | 83.37(8.22 to 308.78) | -0.02 (-0.17 to 0.12) |
| San Marino | 11.39(0.6 to 38.87) | 15.19(0.8 to 53.12) | 35.6(1.85 to 121.46) | 24.17(1.2 to 83.05) | -1.3 (-1.51 to -1.09) |
| Sao Tome and Principe | 81.25(3.06 to 305.53) | 152.36(5.12 to 566.29) | 125.12(4.91 to 474.13) | 136.18(4.83 to 508.94) | 0.25 (0.08 to 0.41) |
| Saudi Arabia | 3871.86(406.34 to 15511.63) | 10138.97(976.77 to 39089.01) | 59.25(7.25 to 241.39) | 44.4(5.4 to 175.97) | -0.86 (-1.01 to -0.71) |
| Senegal | 4218.84(146.36 to 16166.85) | 8327.35(276.31 to 30867.61) | 124.89(4.51 to 476.58) | 107.71(3.74 to 396.19) | -0.29 (-0.44 to -0.14) |
| Serbia | 104605.05(44933.96 to 170137.91) | 72967.15(23778.87 to 136527.7) | 985.08(418.33 to 1604.24) | 458.84(146.26 to 857.39) | -3.17 (-3.42 to -2.91) |
| Seychelles | 209.61(53.2 to 406.57) | 176.87(17.16 to 400.49) | 372.26(91.7 to 726.77) | 161(16.12 to 364) | -3.01 (-3.31 to -2.71) |
| Sierra Leone | 2755.52(90.1 to 10741.15) | 5210.12(156.01 to 19787.23) | 140.97(4.72 to 542.01) | 136.59(4.24 to 510.87) | 0.22 (0.06 to 0.37) |
| Singapore | 5913.04(1271.21 to 11453.59) | 4713.28(745.86 to 10162.55) | 264.67(55.28 to 513.7) | 59.37(9.25 to 128.53) | -5.63 (-5.84 to -5.41) |
| Slovakia | 33382.3(14986.57 to 53455.03) | 19830.07(6435.2 to 36545.37) | 562.15(251.46 to 898.79) | 216.92(70.88 to 397.33) | -3.36 (-3.46 to -3.25) |
| Slovenia | 11745.38(4911.68 to 19766.83) | 5482.32(1718.41 to 10458.35) | 483.94(203.26 to 816.9) | 123.64(38.61 to 235.03) | -5.19 (-5.48 to -4.9) |
| Solomon Islands | 723.7(81.13 to 1933.48) | 1646.46(117.25 to 4594.66) | 568.33(68.17 to 1423.25) | 570.54(44.55 to 1490.64) | -0.14 (-0.26 to -0.02) |
| Somalia | 14802.18(729.28 to 40398.29) | 20174.18(592.38 to 62336.79) | 535.31(30.23 to 1395.05) | 305.1(8.89 to 902.19) | -2 (-2.1 to -1.9) |
| South Africa | 23954.37(966.78 to 84706.73) | 25500.54(1671.5 to 103794.98) | 100.74(4.39 to 361.63) | 55.32(3.93 to 228.12) | -2.21 (-2.66 to -1.75) |
| South Sudan | 9083.79(521.08 to 23323.45) | 6907.24(181.02 to 20809.7) | 376.19(22.73 to 949.79) | 189.49(5.01 to 550.2) | -2.55 (-2.71 to -2.4) |
| Spain | 16754.77(1852.17 to 64738.3) | 13742.49(1489.25 to 46429.71) | 32.85(3.5 to 123.87) | 16.82(1.54 to 53.18) | -2.29 (-2.47 to -2.1) |
| Sri Lanka | 35561.67(9809.2 to 65468.17) | 38216.64(4987.43 to 86719.63) | 336.62(87.54 to 626.8) | 149.85(18.87 to 346.36) | -2.7 (-2.9 to -2.5) |
| Sudan | 7715.58(781.08 to 32548.15) | 10476.64(1158.55 to 42838.28) | 76.65(8.68 to 327.95) | 51.62(6.14 to 210.23) | -1.46 (-1.52 to -1.4) |
| Suriname | 285.28(14.15 to 981.76) | 554.27(26.3 to 1924.45) | 109.92(5.43 to 374.49) | 91.26(4.4 to 319.28) | -1.05 (-1.45 to -0.65) |
| Sweden | 7065.94(419.99 to 21641.14) | 5097.98(303.7 to 16247.5) | 48.69(2.79 to 149.45) | 25.31(1.46 to 78.65) | -2.61 (-2.74 to -2.49) |
| Switzerland | 4083.55(253.42 to 13853.73) | 2625.9(165.98 to 8627.77) | 39.32(2.36 to 131.75) | 15.35(0.91 to 49.33) | -3.36 (-3.57 to -3.15) |
| Syrian Arab Republic | 3639.1(388.3 to 14143.99) | 4874.92(537.04 to 19890.62) | 61.71(7.44 to 246.27) | 38.96(4.86 to 159.42) | -2.17 (-2.45 to -1.89) |
| Taiwan (Province of China) | 42662.82(5448.74 to 97169.07) | 28590.83(2097.07 to 73124.62) | 277.07(37.15 to 622.2) | 73.94(5.45 to 189.79) | -5.01 (-5.3 to -4.72) |
| Tajikistan | 8397.58(1496.37 to 18626.71) | 9921.86(685.2 to 28366.09) | 297.06(53.51 to 664.07) | 216.92(15.79 to 613.35) | -1.23 (-1.45 to -1.01) |
| Thailand | 139713.06(40453.13 to 264796.03) | 149386.45(20377.38 to 343377.44) | 376.46(107.51 to 711.37) | 145.26(19.52 to 334.9) | -3.95 (-4.19 to -3.71) |
| Timor-Leste | 1489.84(397.64 to 2897.34) | 3653.26(533.64 to 8031.49) | 500.73(131.51 to 970.63) | 438.15(63.78 to 974.3) | -0.63 (-0.79 to -0.48) |
| Togo | 1854.88(66.37 to 7267.41) | 5075.39(157.43 to 19437.46) | 136.62(5.15 to 530.72) | 127.76(4.34 to 486.23) | -0.11 (-0.21 to 0) |
| Tokelau | 4.03(0.43 to 10.17) | 2.86(0.23 to 7.35) | 288.42(30 to 727.56) | 211.37(16.42 to 540.19) | -1.31 (-1.46 to -1.17) |
| Tonga | 88.23(10.04 to 217.5) | 113.34(9.05 to 274.8) | 169.5(19.77 to 410.93) | 147.84(11.89 to 357.16) | -0.61 (-0.81 to -0.42) |
| Trinidad and Tobago | 925.12(44.56 to 3107.89) | 1102(46.8 to 3693.15) | 110.05(5.36 to 370.2) | 59.41(2.58 to 199.55) | -2.7 (-2.95 to -2.45) |
| Tunisia | 1838.58(207.01 to 7569.14) | 3394.62(424.25 to 13671.5) | 35.85(4.65 to 145.94) | 27(3.56 to 109.93) | -1.2 (-1.3 to -1.09) |
| Turkey | 7616.12(1396.5 to 32866.65) | 13452.6(2720.88 to 57939.71) | 20.36(4.09 to 88) | 15.18(3.17 to 65.03) | -0.59 (-0.88 to -0.29) |
| Turkmenistan | 6552.06(1215.13 to 14329.22) | 10409.03(794.05 to 28226.62) | 332.77(61.35 to 733.72) | 245.56(19.61 to 674.49) | -1.06 (-1.41 to -0.71) |
| Tuvalu | 26.34(2.9 to 69.41) | 29.29(2.24 to 74.32) | 384.16(43.58 to 1001.92) | 285.61(22.27 to 721.39) | -1.24 (-1.34 to -1.14) |
| Uganda | 22850.1(1238.85 to 57833.39) | 27010.68(827.33 to 82621.8) | 351.61(20.05 to 881.15) | 202.08(6.03 to 598.54) | -2.57 (-2.89 to -2.25) |
| Ukraine | 77470.85(5381.81 to 265519.37) | 65906.25(4323.34 to 225289.89) | 109.32(7.7 to 372.16) | 93.33(5.86 to 316.48) | -1.42 (-1.78 to -1.06) |
| United Arab Emirates | 472.78(37.07 to 1889.42) | 2828.68(210.92 to 10403.26) | 76.22(8.23 to 313.81) | 42.65(4.5 to 164.57) | -2.3 (-2.57 to -2.03) |
| United Kingdom | 39413.41(3488.48 to 140860.13) | 23609.5(2095.97 to 82432.03) | 42.64(3.95 to 151.53) | 18.88(1.69 to 64.24) | -3.1 (-3.34 to -2.85) |
| United Republic of Tanzania | 44823.03(3134.08 to 107130.84) | 70071.31(3186.69 to 175324.81) | 398.38(29.8 to 946.18) | 297.64(14.77 to 721.95) | -0.97 (-1.13 to -0.82) |
| United States of America | 112911.91(7261.48 to 387663.63) | 196781.97(13154.69 to 574968.34) | 36.18(2.28 to 122.81) | 37.95(2.57 to 109.15) | 0.21 (0.06 to 0.37) |
| United States Virgin Islands | 49.06(2.27 to 171.39) | 87.97(4.11 to 300.96) | 58.18(2.74 to 197.3) | 49.24(2.32 to 166.8) | -0.36 (-0.58 to -0.14) |
| Uruguay | 5866.65(368.45 to 15122.76) | 3953.26(211.25 to 10306.68) | 155.5(9.9 to 398.7) | 75.62(4.11 to 198.57) | -2.98 (-3.28 to -2.68) |
| Uzbekistan | 34608.46(6324.93 to 76648.56) | 47756.59(3509.96 to 129729.59) | 301.18(55.02 to 663.52) | 238.06(18.62 to 648.3) | -1.44 (-1.97 to -0.9) |
| Vanuatu | 257.36(28.62 to 667.2) | 681.23(55.82 to 1776.58) | 424.07(46.51 to 1051.1) | 400.9(34.41 to 1047.2) | -0.58 (-0.75 to -0.42) |
| Venezuela (Bolivarian Republic of) | 14809.2(695.08 to 37175.19) | 31745.86(2335.89 to 79147.88) | 143.65(7.05 to 362.01) | 107.26(7.88 to 265.14) | -1.4 (-1.58 to -1.23) |
| Viet Nam | 267760.86(78072.29 to 508291.31) | 414125.91(61765.43 to 875679.46) | 658.79(187.77 to 1252.95) | 440.35(65.71 to 929.31) | -1.18 (-1.32 to -1.03) |
| Yemen | 3778.25(375.28 to 15251.31) | 7661.88(884.67 to 29979.37) | 69.72(8.19 to 284.51) | 51.8(6.92 to 205.29) | -1.28 (-1.37 to -1.19) |
| Zambia | 11034.57(590.86 to 28722.97) | 18814.09(604.7 to 56585.46) | 382.12(22.52 to 987.24) | 298.08(9.29 to 883.96) | -1.17 (-1.33 to -1.02) |
| Zimbabwe | 4420.81(147.09 to 14902.95) | 8771.25(268.75 to 31672.43) | 103.09(3.77 to 363.26) | 116.5(3.96 to 422.2) | 1 (0.7 to 1.3) |

ASDR, age-standard DALYs rate; DALYs, disability-adjusted life years; EAPC, estimated annual percentage change.
